# Abortion Surveillance — United States, 2022

**DOI:** 10.15585/mmwr.ss7307a1

**Published:** 2024-11-28

**Authors:** Stephanie Ramer, Antoinette T. Nguyen, Lisa M. Hollier, Jessica Rodenhizer, Lee Warner, Maura K. Whiteman

**Affiliations:** 1 Division of Reproductive Health, National Center for Chronic Disease Prevention and Health Promotion, CDC

## Abstract

**Problem/Condition:**

CDC conducts abortion surveillance to document the number and characteristics of women obtaining legal induced abortions and the number of abortion-related deaths in the United States.

**Period Covered:**

2022.

**Description of System:**

Each year, CDC requests abortion data from the central health agencies for the 50 states, the District of Columbia, and New York City. For 2022, a total of 48 reporting areas voluntarily provided aggregate abortion data to CDC. Of these, 47 reporting areas provided data each year during 2013–2022. Census and natality data were used to calculate abortion rates (number of abortions per 1,000 women aged 15–44 years) and ratios (number of abortions per 1,000 live births), respectively. Abortion-related deaths from 2021 were assessed as part of CDC’s Pregnancy Mortality Surveillance System (PMSS).

**Results:**

For 2022, a total of 613,383 abortions were reported to CDC from 48 reporting areas. Among 47 reporting areas with data each year during 2013–2022, in 2022, a total of 609,360 abortions were reported, the abortion rate was 11.2 abortions per 1,000 women aged 15–44 years, and the abortion ratio was 199 abortions per 1,000 live births. From 2021 to 2022, the total number of abortions decreased 2% (from 622,108 total abortions), the abortion rate decreased 3% (from 11.6 abortions per 1,000 women aged 15–44 years), and the abortion ratio decreased 2% (from 204 abortions per 1,000 live births). From 2013 to 2022, the total number of reported abortions decreased 5% (from 640,154), the abortion rate decreased 10% (from 12.4 abortions per 1,000 women aged 15–44 years), and the abortion ratio increased 1% (from 198 abortions per 1,000 live births).

In 2022, women in their 20s accounted for more than half of abortions (56.5%). Women aged 20–24 and 25–29 years accounted for the highest percentages of abortions (28.3% and 28.2%, respectively) and had the highest abortion rates (18.1 and 18.7 abortions per 1,000 women aged 20–24 and 25–29 years, respectively). By contrast, adolescents aged <15 years and women aged ≥40 years accounted for the lowest percentages of abortions (0.2% and 3.6%, respectively) and had the lowest abortion rates (0.4 and 2.5 abortions per 1,000 women aged <15 and ≥40 years, respectively). However, abortion ratios were highest among adolescents (aged ≤19 years) and lowest among women aged 30–39 years.

From 2021 to 2022, abortion rates decreased among women aged ≥20 years and did not change among adolescents (aged ≤19 years). Abortion rates decreased from 2013 to 2022 among all age groups, except women aged 30–34 years for whom it increased. The decrease in the abortion rate from 2013 to 2022 was highest among adolescents compared with other age groups. From 2021 to 2022, abortion ratios increased for adolescents and decreased among women aged ≥20 years. From 2013 to 2022, abortion ratios increased among adolescents and women aged 20–34 years and decreased among women aged ≥35 years.

In 2022, the majority (78.6%) of abortions were performed at ≤9 weeks’ gestation, and nearly all (92.8%) were performed at ≤13 weeks’ gestation. During 2013–2022, the percentage of abortions performed at >13 weeks’ gestation remained low (≤8.7%). In 2022, the highest percentage of abortions were performed by early medication abortion at ≤9 weeks’ gestation (53.3%), followed by surgical abortion at ≤13 weeks’ gestation (35.5%), surgical abortion at >13 weeks’ gestation (6.9%), and medication abortion at >9 weeks’ gestation (4.3%); all other methods were uncommon (<0.1%). Among those that were eligible (≤9 weeks’ gestation), 70.2% of abortions were early medication abortions. In 2021, the most recent year for which PMSS data were reviewed for pregnancy-related deaths, five women died as a result of complications from legal induced abortions.

**Interpretation:**

Among the 47 areas that reported data continuously during 2013–2022, overall decreases were observed over this time in the number and rate of reported abortions and an increase was observed in the abortion ratio; in addition, from 2021 to 2022, decreases of 2%–3% were observed across all measures.

**Public Health Action:**

Abortion surveillance can be used to help evaluate programs aimed at promoting equitable access to patient-centered quality contraceptive services in the United States to reduce unintended pregnancies.

## Introduction

This report summarizes data on legal induced abortions for 2022 that were provided voluntarily to CDC by the central health agencies of 48 reporting areas (46 states, the District of Columbia, and New York City, excluding California, Maryland, New Hampshire, and New Jersey) and comparisons over time for the 47 reporting areas that reported each year during 2013–2022 (46 states and New York City). This report also summarizes abortion-related deaths reported voluntarily to CDC for 2021 as part of the Pregnancy Mortality Surveillance System (PMSS). Since 1969, CDC has conducted abortion surveillance to document the number and characteristics of women obtaining legal induced abortions in the United States. After nationwide legalization of abortion in 1973, the total number, rate (number of abortions per 1,000 women aged 15–44 years), and ratio (number of abortions per 1,000 live births) of reported abortions increased rapidly, reaching the highest levels in the 1980s, before decreasing at a slow yet steady pace (*1**,**2*). During 2006–2008, a break occurred in the previously sustained pattern of decrease (*3**,**4*), although this break was followed in subsequent years by even greater decreases (*5**,**6*). However, from 2018 to 2019, increases of 1%–3% were observed across all measures (*7*). From 2019 to 2020, decreases were observed in the number and rate of reported abortions; however, a 2% increase was observed in the abortion ratio (*8*). From 2020 to 2021, increases of 4%–5% were observed across all measures (*9*). Abortion incidence and practices have varied over the years and continue to vary across subpopulations (*10**–**15*), highlighting the utility of continued surveillance.

## Methods

### Description of the Surveillance System

Each year, CDC requests aggregate data from the central health agencies of the 50 states, the District of Columbia, and New York City to document the number and characteristics of women obtaining legal induced abortions in the United States. Not all persons who obtain abortions identify as women; the term “women” has been maintained in this report to be consistent with the collection and reporting of denominator data used to calculate abortion rates and ratios. This report contains data for 2022 voluntarily reported to CDC as of July 15, 2024. For the purpose of surveillance, legal induced abortion is defined as an intervention performed within the limits of state and jurisdiction law by a licensed clinician (e.g., a physician, nurse-midwife, nurse practitioner, or physician assistant) intended to terminate a suspected or known intrauterine pregnancy and that does not result in a live birth. This definition excludes management of intrauterine fetal death, early pregnancy failure or loss, ectopic pregnancy, or retained products of conception. All abortions in this report are considered to be legally induced unless stated otherwise.

In most states and jurisdictions, collection of abortion data is facilitated by a legal requirement for hospitals, facilities, or physicians to report abortions to a central health agency (*16*); however, reporting is not complete in all areas, including in certain areas with reporting requirements (*17*). The reporting of abortion data to CDC is voluntary, and many reporting areas have developed their own data collection forms and might not collect or provide all the information requested by CDC. As a result, the level of detail reported to CDC might vary from year to year and by reporting area. To encourage uniform collection of data, the National Association for Public Health Statistics and Information Systems (NAPHSIS), with technical assistance from CDC, developed technical guidance for vital statistics personnel who collect and summarize abortion data within the United States (*18*).

### Variables and Categorization of Data

Each year, CDC sends a suggested template to central health agencies in the United States for compilation of aggregate abortion data among women obtaining legal induced abortions. Aggregate abortion numbers, without individual-level records, are requested for the following variables:

- Age group in years of women obtaining legal induced abortions (<15, 15–19 [age group and by individual year], 20–24, 25–29, 30–34, 35–39, or ≥40)
- Gestational age of pregnancy in completed weeks at the time of abortion (≤6, 7–20 by individual week, or ≥21)
- Race (Black; White; or other including Alaska Native, American Indian, Asian [Indian, Chinese, Filipino, Japanese, Korean, Vietnamese, or other Asian], Native Hawaiian or other Pacific Islander [Native Hawaiian, Guamanian or Chamorro, Samoan, or other Pacific Islander], other races, and multiple races), ethnicity (Hispanic or non-Hispanic), and race by ethnicity
- Method type (surgical abortion, intrauterine instillation, medication [nonsurgical] abortion, or hysterectomy or hysterotomy)
- Marital status (married [including currently married or separated] or unmarried [including never married, widowed, or divorced])
- Number of previous live births (zero, one, two, three, or four or more)
- Number of previous induced abortions (zero, one, two, or three or more)
- Residence (the state, jurisdiction, territory, or foreign country in which the women obtaining the abortion lived, or, if additional details are unavailable, in-reporting area versus out-of-reporting area)

In addition, the template provided by CDC requests that aggregate abortion numbers for certain variables be cross-tabulated by a second variable. The cross-tabulations presented in this report include weeks of gestation separately by method type, by age group, and by race and ethnicity.

Beginning with 2014 data, instead of reporting the clinician’s estimates of gestational age or estimates of gestational age that were based on last menstrual period, certain areas have reported “probable postfertilization age,” “clinician’s estimate of gestation based on date of conception,” and “probable gestational age” to CDC. To facilitate consistency between data reported as postfertilization age and the data collection practices for gestational age recommended by CDC’s National Center for Health Statistics (*19*), 2 weeks were added to probable postfertilization age. This method was used to account for time after last menstrual period until ovulation in a standard 28-day cycle because fertilization occurs around the time of ovulation (*20*). No modifications were made to data reported as clinician’s estimate of gestational age that was based on date of conception or data reported as probable gestational age.

In this report, medication and surgical abortions are further categorized by gestational age when available in the categories reported to CDC. Early medication abortion is defined as the administration of medications (typically mifepristone followed by misoprostol) to induce an abortion at ≤9 completed weeks’ gestation, consistent with U.S. Food and Drug Administration (FDA) labeling for mifepristone that was implemented in 2016 (*21*). CDC collects information only on the estimated number of weeks (not days) of gestation and acknowledges the conventional use of completed weeks of gestation to describe pregnancy duration; therefore, CDC’s category of ≤9 weeks’ gestation includes abortions through 9 weeks and 6 days. Medications (typically serial prostaglandins, sometimes administered after mifepristone) also might be used to induce an abortion at >9 weeks’ gestation. Surgical abortions, which include uterine aspiration (i.e., dilation and curettage, aspiration curettage, suction curettage, manual vacuum aspiration, menstrual extraction, or sharp curettage) and dilation and evacuation procedures, are categorized as having been performed at ≤13 weeks’ gestation or at >13 weeks’ gestation because of differences in surgical technique at these gestational ages (*22*). Finally, because intrauterine instillation is unlikely to be performed early in gestation (*23*), abortions reported to have been performed by intrauterine instillation at ≤12 weeks’ gestation are excluded from calculation of the percentage of abortions by known method type and are grouped with unknown type.

### Measures of Abortion

Four measures of abortion are presented in this report: 1) the number of abortions in a specific population, 2) the percentage of abortions by selected characteristics, 3) the abortion rate (number of abortions per 1,000 women within a specific population), and 4) the abortion ratio (number of abortions per 1,000 live births within a specific population). Abortion rates adjust for differences in population size. Abortion ratios measure the relative number of pregnancies in a specific population that end in abortion compared with live birth.

U.S. Census Bureau estimates of the resident female population were used as the denominator for calculating abortion rates (*24**–**33*). Overall abortion rates were calculated from the population of women aged 15–44 years living in the reporting areas that provided continuously reported data. For adolescents aged <15 years, abortion rates were calculated using the number of adolescents aged 13–14 years as the denominator; for women aged ≥40 years, abortion rates were calculated using the number of women aged 40–44 years as the denominator. For the calculation of abortion ratios, live birth data were obtained from CDC natality files and included births to women of all ages living in the reporting areas that provided abortion data (*34**,**35*). For calculation of the total abortion rates and total abortion ratios only, women with unknown data on selected characteristics (e.g., age, race and ethnicity, and marital status) were distributed according to the distribution of abortions among those with known information on the characteristic. For calculation of totals only, abortions for women with an unknown gestational age of pregnancy but known method type were distributed according to the distribution of abortions among those with known information on method type by gestational age to the following categories: surgical, ≤13 weeks’ gestation; surgical, >13 weeks’ gestation; medication, ≤9 weeks’ gestation; and medication, >9 weeks’ gestation.

### Data Presentation and Analysis

This report provides aggregate and reporting area–specific abortion numbers, rates, and ratios for the 48 areas that reported to CDC for 2022, which excluded California, Maryland, New Hampshire, and New Jersey. In addition, this report describes characteristics of women who obtained abortions in 2022. The data in this report are presented by the reporting area in which the abortions were performed.

The completeness and quality of data received vary by year, by variable, and by reporting area; this report only describes the characteristics of women obtaining abortions in reporting areas that met CDC reporting standards (i.e., reported at least 20 abortions overall, provided data categorized in accordance with requested variables, and had <15% unknown values for a specific characteristic). Cells with a numerical value in the range of 1–4 and cells that would allow for calculation of these values have been suppressed in this report to maintain confidentiality in tables presented by reporting area of occurrence.

The percentage change in abortion measures (number, rate, and ratio of reported abortions) from the most recent past year (2021 to 2022) and during the 10-year period of analysis (2013–2022) were calculated for the 47 areas that reported every year during 2013–2022. The percentage change was also calculated and reported for abortions by age group, weeks of gestation, and early medication abortions (≤9 completed weeks’ gestation) for areas that met reporting standards for these variables every year during 2013–2022. As a result, aggregate measures for 2022 in percentage change analyses might differ from the point estimates reported for 2022. These data describe the percentage changes in abortion measures over time and abortion measures among groups for each characteristic. No statistical testing was performed. Comparisons do not imply statistical significance, and lack of comment regarding the difference between values does not imply that no statistically significant difference exists.

### Abortion Mortality

CDC has reported data on abortion-related deaths periodically since information on abortion mortality first was included in the 1972 abortion surveillance report (*9**,**36*). An abortion-related death is defined as a death resulting from a direct complication of an abortion (legal or illegal), an indirect complication caused by a chain of events initiated by an abortion, or an aggravation of a pre-existing condition by the physiologic effects of abortion. An abortion is categorized as legal when it is performed by a licensed clinician within the limits of state or jurisdiction law.

Since 1987, CDC has monitored abortion-related deaths through PMSS, which includes data from all 50 states, the District of Columbia, and New York City (*37*). Sources of data to identify abortion-related deaths have included state or jurisdiction vital records; media reports, including computerized searches of full-text newspaper and other media databases; and individual case reports by public health agencies, including maternal mortality review committees, and any additional data when available. For each death that is possibly related to abortion, CDC requests clinical records and autopsy reports. Two medical epidemiologists independently review these reports to determine the cause of death and whether the death was abortion related. Discrepancies are discussed and resolved by consensus. Each death is categorized by abortion type as legal induced, illegal induced, spontaneous, or unknown type.

This report provides PMSS data on induced abortion–related deaths that occurred in 2021, the most recent year for which PMSS data are available. For 1998–2021, abortion surveillance data reported to CDC cannot be used alone to calculate national case-fatality rates for legal induced abortions (number of legal induced abortion–related deaths per 100,000 reported legal induced abortions in the United States) because nine reporting areas did not report abortion data every year during this period (Alaska, 1998–2000; California, 1998–2021; the District of Columbia, 2016; Louisiana, 2005; Maryland, 2007–2021; New Hampshire, 1998–2021; New Jersey, 2021; Oklahoma, 1998–1999; and West Virginia, 2003–2004). Thus, denominator data for calculation of national legal induced abortion case-fatality rates for 1998–2021 were obtained from a published report by the Guttmacher Institute that includes estimated total numbers of abortions in the United States from a national survey of abortion-providing facilities (*38*). For 2021, denominator data for 2020 were used, the most recent year for which data from the Guttmacher Institute are available. Because rates determined on the basis of a numerator <20 are unstable (*39*), national case-fatality rates for legal induced abortion were calculated for consecutive 5-year periods during 1973–2012 and then for a consecutive 9-year period during 2013–2021.

## Results

### Total Abortions Reported to CDC by Occurrence

Among the 48 reporting areas that provided data for 2022, a total of 613,383 abortions were reported (Table 1). Of these abortions, 609,360 were from 47 reporting areas that provided data every year during 2013–2022. In 2022, these continuously reporting areas had an abortion rate of 11.2 abortions per 1,000 women aged 15–44 years and an abortion ratio of 199 abortions per 1,000 live births. Among these 47 reporting areas, from 2021 to 2022, the total number of reported abortions decreased 2% (from 622,108 total abortions), the abortion rate decreased 3% (from 11.6 abortions per 1,000 women aged 15–44 years), and the abortion ratio decreased 2% (from 204 abortions per 1,000 live births). From 2013 to 2022, the total number of reported abortions decreased 5% (from 640,154), the abortion rate decreased 10% (from 12.4 abortions per 1,000 women aged 15–44 years), and the abortion ratio increased 1% (from 198 abortions per 1,000 live births) (Figure).

**TABLE 1:** Number, rate,* and ratio^†^ of reported abortions — selected reporting areas, United States, 2013–2022

Year	Selected reporting areas^§^	Continuously reporting areas^¶^		
	No.	No.	Rate	Ratio
2013	664,435	640,154	12.4	198
2014	652,639	625,668	12.0	190
2015	638,169	613,911	11.8	187
2016	623,471	599,001	11.5	184
2017	612,719	587,611	11.2	184
2018	619,591	591,884	11.2	188
2019	629,898	603,168	11.4	194
2020	620,327	592,939	11.1	197
2021	625,978	622,108	11.6	204
2022	613,383	609,360	11.2	199

* Number of abortions per 1,000 women aged 15–44 years.

^†^ Number of abortions per 1,000 live births.

^§^ For each specific year, excludes reporting areas that did not report that year’s abortion numbers to CDC: California (2013–2022), District of Columbia (2016), Maryland (2013–2022), New Hampshire (2013–2022), and New Jersey (2021–2022).

^¶^ For all years, excludes reporting areas that did not report abortion numbers every year during the analysis period: California, District of Columbia, Maryland, New Hampshire, and New Jersey.

**FIGURE:**
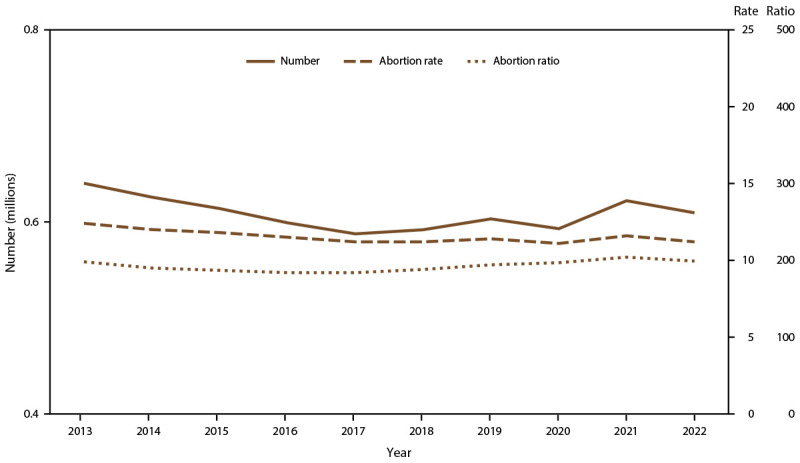
Number, rate,* and ratio^†^ of abortions performed, by year — selected reporting areas,^§^ United States, 2013–2022 * Number of abortions per 1,000 women aged 15–44 years. ^†^ Number of abortions per 1,000 live births. ^§^ Data are for 47 reporting areas; excludes California, District of Columbia, Maryland, New Hampshire, and New Jersey.

In 2022, reporting areas of occurrence had wide variation in abortion rates (from 0.1 to 28.8 abortions per 1,000 women aged 15–44 years in Missouri and New Mexico, respectively) and abortion ratios (from one to 543 abortions per 1,000 live births in Missouri and New Mexico, respectively) (Table 2). The percentage of abortions obtained by out-of-area residents also varied by reporting area (from 0.8% in Arizona to 68.8% in Kansas).

**TABLE 2:** Number, rate,* and ratio^†^ of reported abortions, by reporting area of occurrence and number of abortions obtained by out-of-area residents^§^ — United States, 2022^¶^

Area	Abortions reported by area of occurrence**			Abortions obtained by out-of-area residents
	No.	Rate	Ratio	No. (%)
Alabama	3,777	3.8	65	794 (21.0)
Alaska	1,247	8.6	133	15 (1.2)
Arizona	11,530	8.1	147	97 (0.8)
Arkansas	1,621	2.8	46	437 (27.0)
Colorado	14,154	11.7	227	3,924 (27.7)
Connecticut	11,121	16.0	315	523 (4.7)
Delaware	2,445	13.0	226	254 (10.4)
District of Columbia	4,023	22.4	498	2,696 (67.0)
Florida	82,581	20.5	368	6,721 (8.1)
Georgia	38,820	17.2	308	8,005 (20.6)
Hawaii	1,970	7.5	127	45 (2.3)
Idaho	1,027	2.7	46	49 (4.8)
Illinois	56,457	22.8	440	16,849 (29.8)
Indiana	9,530	7.1	120	1,828 (19.2)
Iowa	4,068	6.7	111	759 (18.7)
Kansas	12,317	21.6	358	8,475 (68.8)
Kentucky	2,550	3.0	49	419 (16.4)
Louisiana	4,570	5.0	81	1,662 (36.4)
Maine	2,225	9.1	184	124 (5.6)
Massachusetts	17,757	12.6	259	920 (5.2)
Michigan	30,120	15.9	294	2,758 (9.2)
Minnesota	12,175	11.1	190	2,009 (16.5)
Mississippi	2,286	3.9	66	648 (28.3)
Missouri	88	0.1	1	16 (18.2)
Montana	1,702	8.1	152	164 (9.6)
Nebraska	2,547	6.7	105	532 (20.9)
Nevada	11,957	19.2	360	1,578 (13.2)
New Mexico	11,735	28.8	543	7,323 (62.4)
New York	72,710	18.7	350	4,183 (5.8)^††^
New York City	46,373	26.2	498	4,463 (9.6)
New York State	26,337	12.5	230	2,052 (7.8)
North Carolina	40,037	19.0	329	11,716 (29.3)
North Dakota	754	4.9	79	222 (29.4)
Ohio	18,499	8.3	144	1,287 (7.0)
Oklahoma	2,160	2.7	45	1,257 (58.2)
Oregon	8,672	10.4	220	1,036 (11.9)
Pennsylvania	34,838	14.2	267	3,311 (9.5)
Rhode Island	1,383	6.4	135	193 (14.0)
South Carolina	7,276	7.2	126	1,709 (23.5)
South Dakota	137	0.8	12	14 (10.2)
Tennessee	6,452	4.6	78	1,333 (20.7)
Texas	17,514	2.8	45	302 (1.7)
Utah	3,824	5.1	84	215 (5.6)
Vermont	1,108	9.2	208	217 (19.6)
Virginia^§§^	16,722	9.7	175	2,454 (14.7)
Washington	20,193	12.9	242	1,440 (7.1)
West Virginia	831	2.7	49	182 (21.9)
Wisconsin	3,333	3.0	56	54 (1.6)
Wyoming	540	4.9	89	166 (30.7)
**Total**	**613,383**	**NA**	**NA**	**NA**

**Abbreviation:** NA = not applicable.

* Number of abortions per 1,000 women aged 15–44 years.

^†^ Number of abortions per 1,000 live births.

^§^ Additional details on the reporting area in which abortions were provided, cross-tabulated by the area of residence, are available at https://www.cdc.gov/reproductive-health/data-statistics/abortion-surveillance-findings-reports.html.

^¶^ Data from 48 reporting areas; excludes four reporting areas (California, Maryland, New Hampshire, and New Jersey) that did not report.

** The total abortions include those with known and unknown residence status.

^††^ Residents of New York State who had abortions in New York City and residents of New York City who had abortions in New York State were excluded from the number and percentage of abortions obtained by out-of-area residents in New York.

^§§^ Data reported from facilities physically situated in state only.

### Age Group, Race and Ethnicity, and Marital Status

Among the 48 areas that reported abortion numbers by women’s age for 2022, women in their 20s accounted for more than half of abortions (56.5%) (Table 3). Women aged 20–24 and 25–29 years accounted for the highest percentages of abortions (28.3% and 28.2%, respectively) and had the highest abortion rates (18.1 and 18.7 abortions per 1,000 women aged 20–24 and 25–29 years, respectively). By contrast, adolescents aged <15 years and women aged ≥40 years accounted for the lowest percentages of abortions (0.2% and 3.6%, respectively) and had the lowest abortion rates (0.4 and 2.5 abortions per 1,000 adolescents aged <15 and women ≥40 years, respectively). However, abortion ratios were highest among adolescents (800 and 389 abortions per 1,000 live births among those aged <15 years and 15–19 years, respectively) and lowest among women aged ≥25 years (194, 133, 134, and 195 abortions per 1,000 live births among those aged 25–29, 30–34, 35–39 years, and ≥40 years, respectively).

**TABLE 3:** Number and percentage of reported abortions, by known age group and reporting area of occurrence — selected reporting areas,^*^ United States, 2022

Area	Age group, yrs							Total abortions reported by known age
	<15	15–19	20–24	25–29	30–34	35–39	≥40	
	No. (%)^†^	No. (%)	No. (%)	No. (%)	No. (%)	No. (%)	No. (%)	No. (% of all reported abortions)^§^
Alabama	11 (0.3)	366 (9.7)	1,240 (32.8)	1,061 (28.1)	691 (18.3)	300 (7.9)	106 (2.8)	**3,775 (99.9)**
Alaska	5 (0.4)	123 (9.9)	389 (31.2)	320 (25.7)	235 (18.9)	136 (10.9)	38 (3.0)	**1,246 (99.9)**
Arizona	12 (0.1)	1,095 (9.5)	3,562 (30.9)	3,188 (27.6)	2,093 (18.2)	1,165 (10.1)	415 (3.6)	**11,530 (100.0)**
Arkansas	9 (0.6)	167 (10.3)	500 (30.9)	479 (29.6)	302 (18.7)	125 (7.7)	37 (2.3)	**1,619 (99.9)**
Colorado	47 (0.3)	1,439 (10.2)	4,434 (31.4)	3,801 (26.9)	2,582 (18.3)	1,345 (9.5)	483 (3.4)	**14,131 (99.8)**
Connecticut	22 (0.2)	1,009 (9.1)	3,036 (27.3)	3,021 (27.2)	2,322 (20.9)	1,290 (11.6)	410 (3.7)	**11,110 (99.9)**
Delaware	6 (0.2)	238 (9.7)	721 (29.5)	685 (28.0)	474 (19.4)	243 (9.9)	78 (3.2)	**2,445 (100.0)**
District of Columbia	12 (0.3)	427 (10.6)	1,147 (28.5)	1,182 (29.4)	764 (19.0)	364 (9.1)	124 (3.1)	**4,020 (99.9)**
Florida	137 (0.2)	5,756 (7.0)	22,427 (27.2)	23,231 (28.2)	17,684 (21.5)	9,718 (11.8)	3,369 (4.1)	**82,322 (99.7)**
Georgia	74 (0.2)	2,750 (7.1)	10,684 (27.5)	11,634 (30.0)	8,339 (21.5)	3,957 (10.2)	1,382 (3.6)	**38,820 (100.0)**
Hawaii	—^¶^	181 (9.2)	552 (28.0)	526 (26.7)	382 (19.4)	228 (11.6)	—^¶^	**1,970 (100.0)**
Idaho	—^¶^	125 (12.2)	349 (34.0)	277 (27.0)	155 (15.1)	94 (9.2)	—^¶^	**1,027 (100.0)**
Illinois**	75 (0.2)	3,303 (8.5)	11,189 (28.8)	11,593 (29.9)	7,827 (20.2)	3,698 (9.5)	1,128 (2.9)	**38,813 (99.6)**
Indiana	22 (0.2)	840 (8.8)	2,910 (30.5)	2,677 (28.1)	1,849 (19.4)	893 (9.4)	339 (3.6)	**9,530 (100.0)**
Iowa	13 (0.3)	420 (10.3)	1,277 (31.4)	1,048 (25.8)	746 (18.4)	430 (10.6)	129 (3.2)	**4,063 (99.9)**
Kansas	35 (0.3)	1,159 (9.4)	3,908 (31.7)	3,322 (27.0)	2,232 (18.1)	1,278 (10.4)	383 (3.1)	**12,317 (100.0)**
Kentucky	10 (0.4)	201 (7.9)	831 (32.6)	691 (27.1)	498 (19.5)	245 (9.6)	74 (2.9)	**2,550 (100.0)**
Louisiana	20 (0.4)	425 (9.3)	1,379 (30.2)	1,250 (27.4)	904 (19.8)	476 (10.4)	116 (2.5)	**4,570 (100.0)**
Maine	6 (0.3)	210 (9.5)	596 (26.9)	589 (26.6)	457 (20.6)	271 (12.2)	89 (4.0)	**2,218 (99.7)**
Massachusetts	32 (0.2)	1,392 (7.8)	4,622 (26.0)	4,710 (26.5)	3,897 (22.0)	2,232 (12.6)	863 (4.9)	**17,748 (99.9)**
Michigan	85 (0.3)	2,328 (7.8)	8,297 (27.7)	8,766 (29.3)	6,742 (22.5)	2,848 (9.5)	867 (2.9)	**29,933 (99.4)**
Minnesota	26 (0.2)	1,117 (9.2)	3,431 (28.2)	3,277 (26.9)	2,523 (20.7)	1,338 (11.0)	454 (3.7)	**12,166 (99.9)**
Mississippi	7 (0.3)	200 (8.7)	738 (32.3)	705 (30.8)	416 (18.2)	171 (7.5)	49 (2.1)	**2,286 (100.0)**
Missouri	—^¶^	—^¶^	18 (20.5)	21 (23.9)	20 (22.7)	15 (17.0)	10 (11.4)	**88 (100.0)**
Montana	6 (0.4)	189 (11.1)	490 (28.8)	444 (26.1)	313 (18.4)	186 (10.9)	74 (4.3)	**1,702 (100.0)**
Nebraska	7 (0.3)	270 (10.6)	850 (33.4)	676 (26.5)	419 (16.5)	252 (9.9)	73 (2.9)	**2,547 (100.0)**
Nevada^††^	11 (0.1)	964 (8.4)	3,061 (26.7)	3,206 (28.0)	2,410 (21.1)	1,298 (11.3)	494 (4.3)	**11,444 (95.7)**
New Mexico	48 (0.4)	1,234 (10.7)	3,528 (30.7)	3,014 (26.2)	2,048 (17.8)	1,248 (10.9)	368 (3.2)	**11,488 (97.9)**
New York	169 (0.2)	5,941 (8.2)	19,084 (26.3)	20,173 (27.8)	15,544 (21.4)	8,626 (11.9)	3,132 (4.3)	**72,669 (99.9)**
New York City	89 (0.2)	3,465 (7.5)	11,748 (25.3)	13,067 (28.2)	10,140 (21.9)	5,733 (12.4)	2,131 (4.6)	**46,373 (100.0)**
New York State	80 (0.3)	2,476 (9.4)	7,336 (27.9)	7,106 (27.0)	5,404 (20.6)	2,893 (11.0)	1,001 (3.8)	**26,296 (99.8)**
North Carolina	82 (0.2)	3,154 (8.0)	11,508 (29.3)	11,532 (29.4)	7,974 (20.3)	3,864 (9.8)	1,172 (3.0)	**39,286 (98.1)**
North Dakota	6 (0.8)	90 (11.9)	215 (28.5)	191 (25.3)	160 (21.2)	79 (10.5)	13 (1.7)	**754 (100.0)**
Ohio	42 (0.2)	1,494 (8.1)	5,455 (29.5)	5,325 (28.8)	3,791 (20.5)	1,825 (9.9)	567 (3.1)	**18,499 (100.0)**
Oklahoma	12 (0.6)	244 (11.3)	732 (34.0)	574 (26.6)	353 (16.4)	190 (8.8)	50 (2.3)	**2,155 (99.8)**
Oregon	12 (0.1)	894 (10.3)	2,561 (29.5)	2,336 (26.9)	1,636 (18.9)	922 (10.6)	309 (3.6)	**8,670 (100.0)**
Pennsylvania	76 (0.2)	2,724 (7.8)	9,351 (26.8)	10,107 (29.0)	7,591 (21.8)	3,765 (10.8)	1,224 (3.5)	**34,838 (100.0)**
Rhode Island	—^¶^	114 (8.2)	362 (26.2)	368 (26.6)	294 (21.3)	190 (13.7)	—^¶^	**1,383 (100.0)**
South Carolina	19 (0.3)	641 (8.8)	2,072 (28.5)	2,087 (28.7)	1,457 (20.0)	759 (10.4)	241 (3.3)	**7,276 (100.0)**
South Dakota	—^¶^	—^¶^	51 (37.2)	32 (23.4)	25 (18.2)	16 (11.7)	—^¶^	**137 (100.0)**
Tennessee	14 (0.2)	513 (8.0)	1,846 (28.6)	1,979 (30.7)	1,318 (20.4)	584 (9.1)	197 (3.1)	**6,451 (100.0)**
Texas	14 (0.1)	1,242 (7.1)	5,154 (29.4)	5,101 (29.1)	3,566 (20.4)	1,835 (10.5)	602 (3.4)	**17,514 (100.0)**
Utah	8 (0.2)	469 (12.3)	1,313 (34.3)	928 (24.3)	623 (16.3)	350 (9.2)	133 (3.5)	**3,824 (100.0)**
Vermont	—^¶^	100 (9.0)	320 (28.9)	274 (24.7)	243 (21.9)	120 (10.8)	—^¶^	**1,108 (100.0)**
Virginia^§§^	38 (0.2)	1,155 (6.9)	4,515 (27.0)	4,767 (28.5)	3,498 (20.9)	2,007 (12.0)	739 (4.4)	**16,719 (100.0)**
Washington	39 (0.2)	2,063 (10.2)	5,669 (28.1)	5,268 (26.1)	3,884 (19.2)	2,388 (11.8)	871 (4.3)	**20,182 (99.9)**
West Virginia	—^¶^	92 (11.1)	268 (32.3)	232 (28.0)	149 (18.0)	60 (7.2)	—^¶^	**830 (99.9)**
Wisconsin	13 (0.4)	337 (10.1)	1,085 (32.6)	878 (26.3)	619 (18.6)	315 (9.5)	86 (2.6)	**3,333 (100.0)**
Wyoming	0 (0.0)	41 (7.6)	141 (26.1)	143 (26.5)	115 (21.3)	70 (13.0)	30 (5.6)	**540 (100.0)**
**Total**	**1,292 (0.2)**	**49,251 (8.3)**	**167,868 (28.3)**	**167,689 (28.2)**	**122,164 (20.6)**	**63,809 (10.7)**	**21,573 (3.6)**	**593,646 (99.6)^¶¶^**
Abortion rate***	**0.4**	**5.6**	**18.1**	**18.7**	**13.0**	**7.1**	**2.5**	**NA**
**Abortion ratio^†††^**	**800**	**389**	**301**	**194**	**133**	**134**	**195**	**NA**

**Abbreviation:** NA = not applicable.

* Data from 48 reporting areas; excludes four reporting areas (California, Maryland, New Hampshire, and New Jersey) that did not report, did not report by age, or did not meet reporting standards.

^†^ Percentages for the individual component categories might not add to 100% because of rounding.

^§^ Percentage is calculated as the number of abortions reported by known age divided by the sum of abortions reported by known and unknown age. Values ≥99.95% are rounded to 100.0%.

^¶^ Cells with a numerical value in the range of 1–4 and cells that would allow for calculation of these small values have been suppressed.

** Includes residents only.

^††^ For August–December 2022, variable was collected for residents only and reported as unknown for nonresidents.

^§§^ Data reported from facilities physically situated in state only.

^¶¶^ Percentage is based on a total of 613,383 abortions reported among the areas that met reporting standards for age; reporting standards for age were applied to abortions for residents of Illinois only.

*** Number of abortions obtained by women in a specific age group per 1,000 women in that same age group. Adolescents aged 13–14 years were used as the denominator for the group of adolescents aged <15 years, and women aged 40–44 years were used as the denominator for the group of women aged ≥40 years. For the total abortion rate only, abortions for women of unknown age were distributed according to the distribution of abortions among women of known age.

^†††^ Number of abortions obtained by women in a specific age group per 1,000 live births to women in that same age group. For the total abortion ratio only, abortions for women of unknown age were distributed according to the distribution of abortions among women of known age.

Among the 43 reporting areas that provided data each year by women’s age for 2013–2022, the highest percentages of abortions and the highest abortion rates occurred among women aged 20–29 years and the lowest percentages of abortions and lowest abortion rates occurred among adolescents aged <15 years and women aged ≥40 years (Table 4). From 2021 to 2022, abortion rates decreased among women aged ≥20 years and did not change among adolescents aged <15 years and adolescents aged 15–19 years.

**TABLE 4:** Percentage, rate,^*^ and ratio^†^ of reported abortions, by known age group and year — selected reporting areas,^§^ United States, 2013–2022

Age group, yrs	Year										% Change	
	2013	2014	2015	2016	2017	2018	2019	2020	2021	2022	2021 to 2022	2013 to 2022
**Reported abortions by known age (%)**												
<15	0.3	0.3	0.3	0.3	0.2	0.2	0.2	0.2	0.2	0.2	0.0	−33.3
15–19	11.5	10.4	9.8	9.4	9.1	8.8	8.7	8.3	8.2	8.5	3.7	−26.1
20–24	32.7	32.2	31.2	30.1	29.4	28.6	27.9	28.2	28.5	28.5	0.0	−12.8
25–29	25.9	26.7	27.6	28.5	28.9	29.3	29.3	29.3	28.7	28.2	−1.7	8.9
30–34	16.8	17.2	17.7	18.0	18.3	18.8	19.4	19.7	20.2	20.4	1.0	21.4
35–39	9.2	9.6	10.0	10.3	10.5	10.7	10.8	10.6	10.6	10.6	0.0	15.2
≥40	3.6	3.6	3.6	3.6	3.6	3.5	3.7	3.6	3.5	3.6	2.9	0.0
**Abortion rate**												
<15	0.6	0.5	0.5	0.4	0.4	0.4	0.4	0.4	0.4	0.4	0.0	−33.3
15–19	8.2	7.3	6.7	6.2	5.9	5.8	5.8	5.5	5.4	5.4	0.0	−34.1
20–24	21.8	20.8	19.9	19.0	18.3	18.1	18.1	18.1	18.6	17.1	−8.1	−21.6
25–29	18.1	17.9	17.8	17.7	17.3	17.5	17.8	17.7	18.3	17.7	−3.3	−2.2
30–34	11.8	11.6	11.7	11.6	11.5	11.8	12.3	12.1	12.5	12.2	−2.4	3.4
35–39	7.0	7.0	7.0	6.9	6.7	6.8	7.0	6.7	6.8	6.6	−2.9	−5.7
≥40	2.6	2.5	2.5	2.5	2.5	2.5	2.6	2.4	2.4	2.3	−4.2	−11.5
**Abortion ratio**												
<15	771	737	690	724	788	846	858	809	780	796	2.1	3.2
15–19	297	289	286	292	300	315	330	338	366	374	2.2	25.9
20–24	260	253	249	247	248	254	259	273	290	284	−2.1	9.2
25–29	167	164	165	167	170	177	182	189	189	182	−3.7	9.0
30–34	122	116	115	113	114	119	125	127	127	125	−1.6	2.5
35–39	149	145	141	137	135	136	138	135	132	125	−5.3	−16.1
≥40	247	240	230	221	213	209	216	207	198	183	−7.6	−25.9
**Total no.^¶^**	**544,986**	**531,041**	**520,602**	**508,565**	**497,249**	**500,974**	**508,955**	**502,481**	**514,695**	**498,095**	**NA**	**NA**

**Abbreviation:** NA = not applicable.

* Number of abortions obtained by women in a specific age group per 1,000 women in that same age group. Adolescents aged 13–14 years were used as the denominator for the group of adolescents aged <15 years, and women aged 40–44 years were used as the denominator for the group of women aged ≥40 years. Abortions for women of unknown age were distributed according to the distribution of abortions among women of known age.

^†^ Number of abortions obtained by women in a specific age group per 1,000 live births to women in that same age group. Abortions for women of unknown age were distributed according to the distribution of abortions among women of known age.

^§^ Data from 43 reporting areas; excludes nine reporting areas (California, District of Columbia, Florida, Maine, Maryland, New Hampshire, New Jersey, Tennessee, and Wyoming) that did not report, did not report by age, or did not meet reporting standards for ≥1 year.

^¶^ By year, the total number of abortions represent 99.5%–99.7% of all abortions reported to CDC among the areas that met reporting standards for age during 2013–2022; reporting standards for age were applied to abortions for residents of Illinois only during 2013–2019 and 2021–2022.

From 2013 to 2022, abortion rates decreased among all age groups, except women aged 30–34 years for whom it increased. The decrease in the abortion rate was highest among adolescents (33% and 34% for adolescents aged <15 and 15–19 years, respectively) compared with any other age group. From 2021 to 2022, abortion ratios increased for adolescents aged <15 years and 15–19 years and decreased among women aged ≥20 years. From 2013 to 2022, abortion ratios increased among adolescents aged ≤19 years and women aged 20–34 years and decreased among women aged ≥35 years.

Among the 45 areas that reported age by individual year among adolescents for 2022, adolescents aged 18–19 years accounted for the highest percentage (71.6%) of adolescent abortions and had the highest abortion rates (8.6 and 12.4 abortions per 1,000 adolescents aged 18 and 19 years, respectively) (Table 5). Adolescents aged <15 years accounted for the lowest percentage of adolescent abortions (2.6%) and had the lowest abortion rate (0.4 abortions per 1,000 adolescents aged 13–14 years). The abortion ratio for adolescents was highest among those aged <15 years (773 abortions per 1,000 live births) and was lowest among those aged 17–19 years (363, 414, and 343 abortions per 1,000 live births among adolescents aged 17, 18, and 19 years, respectively).

**TABLE 5:** Number and percentage of reported abortions among adolescents, by known age and reporting area of occurrence — selected reporting areas,* United States, 2022

Area	Age, yrs						Total no.
	<15	15	16	17	18	19	
	No. (%)^†^	No. (%)	No. (%)	No. (%)	No. (%)	No. (%)	
Alabama	11 (2.9)	24 (6.4)	33 (8.8)	39 (10.3)	109 (28.9)	161 (42.7)	**377**
Alaska	5 (3.9)	7 (5.5)	10 (7.8)	18 (14.1)	33 (25.8)	55 (43.0)	**128**
Arizona	12 (1.1)	39 (3.5)	71 (6.4)	129 (11.7)	369 (33.3)	487 (44.0)	**1,107**
Arkansas	9 (5.1)	8 (4.5)	11 (6.3)	27 (15.3)	54 (30.7)	67 (38.1)	**176**
Colorado	47 (3.2)	63 (4.2)	139 (9.4)	211 (14.2)	434 (29.2)	592 (39.8)	**1,486**
Delaware	6 (2.5)	8 (3.3)	24 (9.8)	43 (17.6)	68 (27.9)	95 (38.9)	**244**
District of Columbia	12 (2.7)	14 (3.2)	61 (13.9)	103 (23.5)	91 (20.7)	158 (36.0)	**439**
Florida	137 (2.3)	208 (3.5)	423 (7.2)	708 (12.0)	1,730 (29.4)	2,687 (45.6)	**5,893**
Georgia	74 (2.6)	122 (4.3)	191 (6.8)	346 (12.3)	840 (29.7)	1,251 (44.3)	**2,824**
Hawaii	—^§^	—^§^	—^§^	—^§^	—^§^	—^§^	**—^§^**
Idaho	—^§^	—^§^	—^§^	—^§^	—^§^	—^§^	**—^§^**
Indiana	22 (2.6)	37 (4.3)	70 (8.1)	100 (11.6)	278 (32.3)	355 (41.2)	**862**
Iowa	13 (3.0)	22 (5.1)	30 (6.9)	74 (17.1)	119 (27.5)	175 (40.4)	**433**
Kansas	35 (2.9)	60 (5.0)	78 (6.5)	127 (10.6)	371 (31.1)	523 (43.8)	**1,194**
Kentucky	10 (4.7)	9 (4.3)	14 (6.6)	35 (16.6)	58 (27.5)	85 (40.3)	**211**
Louisiana	20 (4.5)	29 (6.5)	45 (10.1)	44 (9.9)	110 (24.7)	197 (44.3)	**445**
Maine	6 (2.8)	13 (6.0)	15 (6.9)	36 (16.7)	60 (27.8)	86 (39.8)	**216**
Massachusetts	32 (2.2)	54 (3.8)	129 (9.1)	195 (13.7)	399 (28.0)	615 (43.2)	**1,424**
Michigan	85 (3.5)	98 (4.1)	196 (8.1)	338 (14.0)	702 (29.1)	994 (41.2)	**2,413**
Minnesota	26 (2.3)	45 (3.9)	87 (7.6)	148 (12.9)	355 (31.1)	482 (42.2)	**1,143**
Mississippi	7 (3.4)	14 (6.8)	18 (8.7)	25 (12.1)	56 (27.1)	87 (42.0)	**207**
Missouri	—^§^	—^§^	—^§^	—^§^	—^§^	—^§^	**—^§^**
Montana	6 (3.1)	8 (4.1)	16 (8.2)	27 (13.8)	53 (27.2)	85 (43.6)	**195**
Nebraska	7 (2.5)	13 (4.7)	21 (7.6)	45 (16.2)	82 (29.6)	109 (39.4)	**277**
Nevada^¶^	11 (1.1)	50 (5.1)	75 (7.7)	156 (16.0)	272 (27.9)	411 (42.2)	**975**
New Mexico	48 (3.7)	66 (5.1)	115 (9.0)	208 (16.2)	352 (27.5)	493 (38.5)	**1,282**
New York	169 (2.8)	272 (4.5)	581 (9.5)	957 (15.7)	1,688 (27.6)	2,443 (40.0)	**6,110**
New York City	89 (2.5)	164 (4.6)	334 (9.4)	547 (15.4)	973 (27.4)	1,447 (40.7)	**3,554**
New York State	80 (3.1)	108 (4.2)	247 (9.7)	410 (16.0)	715 (28.0)	996 (39.0)	**2,556**
North Carolina	82 (2.5)	146 (4.5)	262 (8.1)	393 (12.1)	1,016 (31.4)	1,337 (41.3)	**3,236**
North Dakota	6 (6.3)	—^§^	—^§^	13 (13.5)	33 (34.4)	34 (35.4)	**96**
Ohio	42 (2.7)	76 (4.9)	124 (8.1)	213 (13.9)	440 (28.6)	641 (41.7)	**1,536**
Oklahoma	12 (4.7)	9 (3.5)	17 (6.6)	21 (8.2)	97 (37.9)	100 (39.1)	**256**
Oregon	12 (1.3)	28 (3.1)	68 (7.5)	138 (15.2)	292 (32.2)	368 (40.6)	**906**
Pennsylvania	76 (2.7)	147 (5.3)	207 (7.4)	331 (11.8)	828 (29.6)	1,211 (43.3)	**2,800**
Rhode Island	—^§^	—^§^	—^§^	—^§^	—^§^	—^§^	**—^§^**
South Carolina	19 (2.9)	28 (4.2)	50 (7.6)	125 (18.9)	211 (32.0)	227 (34.4)	**660**
South Dakota	—^§^	—^§^	—^§^	—^§^	—^§^	—^§^	**—^§^**
Tennessee	14 (2.7)	16 (3.0)	47 (8.9)	58 (11.0)	158 (30.0)	234 (44.4)	**527**
Texas	14 (1.1)	42 (3.3)	76 (6.1)	145 (11.5)	362 (28.8)	617 (49.1)	**1,256**
Utah	8 (1.7)	18 (3.8)	32 (6.7)	51 (10.7)	165 (34.6)	203 (42.6)	**477**
Vermont	—^§^	—^§^	—^§^	—^§^	—^§^	—^§^	**—^§^**
Virginia**	38 (3.2)	54 (4.5)	79 (6.6)	142 (11.9)	326 (27.3)	554 (46.4)	**1,193**
Washington	39 (1.9)	94 (4.5)	199 (9.5)	317 (15.1)	594 (28.3)	859 (40.9)	**2,102**
West Virginia	—^§^	—^§^	—^§^	—^§^	—^§^	—^§^	**—^§^**
Wyoming	0 (0.0)	—^§^	—^§^	—^§^	15 (36.6)	22 (53.7)	**41**
**Total**	**1,182 (2.6)**	**1,973 (4.3)**	**3,683 (8.0)**	**6,178 (13.5)**	**13,408 (29.3)**	**19,360 (42.3)**	**45,784**
**Abortion rate^††^**	**0.4**	**1.2**	**2.3**	**4.0**	**8.6**	**12.4**	**NA**
**Abortion ratio^§§^**	**773**	**528**	**435**	**363**	**414**	**343**	**NA**

**Abbreviation:** NA = not applicable.

* Data from 45 reporting areas; excludes seven reporting areas (California, Connecticut, Illinois, Maryland, New Hampshire, New Jersey, and Wisconsin) that did not report, did not report age among adolescents by individual year, or did not meet reporting standards.

^†^ Percentages for the individual component categories might not add to 100% because of rounding.

^§^ Cells with a numerical value in the range of 1–4 and cells that would allow for calculation of these small values have been suppressed.

^¶^ For August–December 2022, variable was collected for residents only and reported as unknown for nonresidents.

** Data reported from facilities physically situated in state only.

^††^ Number of abortions obtained by women in a specific age group per 1,000 women in that same age group. Adolescents aged 13–14 years were used as the denominator for the group of adolescents aged <15 years. For the total abortion rate only, abortions for women of unknown age were distributed according to the distribution of abortions among women of known age.

^§§^ Number of abortions obtained by women in a specific age group per 1,000 live births to women in that same age group. For the total abortion ratio only, abortions for women of unknown age were distributed according to the distribution of abortions among women of known age.

Among the 32 areas that reported race by ethnicity data for 2022, non-Hispanic White (White) women and non-Hispanic Black (Black) women accounted for the highest percentages of all abortions (31.9% and 39.5%, respectively), and Hispanic women and non-Hispanic women in the other race category accounted for lower percentages (21.2% and 7.3%, respectively) (Table 6). White women had the lowest abortion rate (5.7 abortions per 1,000 women aged 15–44 years) and ratio (106 abortions per 1,000 live births), and Black women had the highest abortion rate (24.4 abortions per 1,000 women aged 15–44 years) and ratio (429 abortions per 1,000 live births).

**TABLE 6:** Number and percentage of reported abortions, by known race and ethnicity and reporting area of occurrence — selected reporting areas,* United States, 2022

Area	Non-Hispanic			Hispanic	Total abortions reported by known race and ethnicity
	Black	White	Other^†^		
	No. (%)^§^	No. (%)	No. (%)	No. (%)	No. (% of all reported abortions)^¶^
Alabama	2,017 (54.0)	907 (24.3)	68 (1.8)	742 (19.9)	**3,734 (98.9)**
Alaska	98 (8.6)	547 (48.0)	467 (41.0)	27 (2.4)	**1,139 (91.3)**
Arizona	1,400 (12.7)	3,699 (33.6)	769 (7.0)	5,127 (46.6)	**10,995 (95.4)**
Delaware	1,104 (45.2)	864 (35.3)	55 (2.2)	422 (17.3)	**2,445 (100.0)**
District of Columbia	2,123 (56.6)	489 (13.0)	436 (11.6)	700 (18.7)	**3,748 (93.2)**
Florida	28,192 (36.3)	21,866 (28.2)	2,845 (3.7)	24,679 (31.8)	**77,582 (93.9)**
Georgia	24,104 (67.6)	6,492 (18.2)	1,687 (4.7)	3,399 (9.5)	**35,682 (91.9)**
Idaho	22 (2.2)	658 (65.7)	40 (4.0)	281 (28.1)	**1,001 (97.5)**
Indiana	3,370 (36.5)	4,188 (45.4)	687 (7.4)	981 (10.6)	**9,226 (96.8)**
Kansas	3,016 (24.7)	4,568 (37.5)	1,152 (9.4)	3,461 (28.4)	**12,197 (99.0)**
Kentucky	928 (36.5)	1,215 (47.8)	133 (5.2)	268 (10.5)	**2,544 (99.8)**
Maine	174 (8.3)	1,775 (84.7)	71 (3.4)	76 (3.6)	**2,096 (94.2)**
Michigan	15,901 (54.2)	10,616 (36.2)	1,525 (5.2)	1,305 (4.4)	**29,347 (97.4)**
Minnesota	2,848 (25.8)	5,198 (47.0)	1,630 (14.7)	1,377 (12.5)	**11,053 (90.8)**
Mississippi	1,767 (77.3)	438 (19.2)	22 (1.0)	59 (2.6)	**2,286 (100.0)**
Missouri	34 (40.5)	40 (47.6)	—**	—**	**84 (95.5)**
Montana	30 (1.8)	1,364 (80.1)	169 (9.9)	139 (8.2)	**1,702 (100.0)**
Nevada^††^	1,908 (18.5)	2,865 (27.8)	799 (7.7)	4,740 (46.0)	**10,312 (86.2)**
New York State	7,007 (31.3)	8,683 (38.8)	1,722 (7.7)	4,991 (22.3)	**22,403 (85.1)**
North Carolina	19,576 (51.3)	9,936 (26.0)	3,100 (8.1)	5,579 (14.6)	**38,191 (95.4)**
North Dakota	119 (16.6)	428 (59.8)	121 (16.9)	48 (6.7)	**716 (95.0)**
Oregon	518 (6.6)	4,272 (54.7)	919 (11.8)	2,101 (26.9)	**7,810 (90.1)**
Rhode Island	202 (15.2)	803 (60.4)	69 (5.2)	255 (19.2)	**1,329 (96.1)**
South Carolina	3,463 (47.6)	2,700 (37.1)	315 (4.3)	795 (10.9)	**7,273 (100.0)**
South Dakota	15 (11.0)	84 (61.8)	26 (19.1)	11 (8.1)	**136 (99.3)**
Tennessee	3,448 (55.3)	2,028 (32.5)	213 (3.4)	548 (8.8)	**6,237 (96.7)**
Texas^§§^	5,194 (30.0)	4,539 (26.2)	922 (5.3)	6,659 (38.5)	**17,314 (98.9)**
Utah	186 (5.1)	1,939 (52.9)	295 (8.0)	1,248 (34.0)	**3,668 (95.9)**
Vermont	43 (4.0)	943 (88.6)	30 (2.8)	48 (4.5)	**1,064 (96.0)**
Virginia^¶¶^	5,386 (34.8)	3,445 (22.2)	4,586 (29.6)	2,075 (13.4)	**15,492 (92.6)**
West Virginia	109 (13.1)	692 (83.3)	—**	—**	**831 (100.0)**
Wyoming	6 (1.2)	361 (74.3)	29 (6.0)	90 (18.5)	**486 (90.0)**
**Total**	**134,308 (39.5)**	**108,642 (31.9)**	**24,932 (7.3)**	**72,241 (21.2)**	340,123 (94.0)***
**Abortion rate^†††^**	**24.4**	**5.7**	**9.4**	**11.6**	**NA**
**Abortion ratio^§§§^**	**429**	**106**	**182**	**165**	**NA**

* Data from 32 reporting areas; excludes 20 reporting areas (Arkansas, California, Colorado, Connecticut, Hawaii, Illinois, Iowa, Louisiana, Maryland, Massachusetts, Nebraska, New Hampshire, New Jersey, New Mexico, New York City, Ohio, Oklahoma, Pennsylvania, Washington, and Wisconsin) that did not report, did not report by race and ethnicity, or did not meet reporting standards.

^†^ Including Alaska Native, American Indian, Asian (Indian, Chinese, Filipino, Japanese, Korean, Vietnamese, or other Asian), Pacific Islander (Native Hawaiian, Guamanian or Chamorro, Samoan, or other Pacific Islander), other races, and multiple races.

^§^ Percentages for the individual component categories might not add to 100% because of rounding.

^¶^ Percentage is calculated as the number of abortions reported by known race and ethnicity divided by the sum of abortions reported by known and unknown race and ethnicity. Values ≥99.95% are rounded to 100.0%.

** Cells with a numerical value in the range of 1–4 and cells that would allow for calculation of these small values have been suppressed.

^††^ For August–December 2022, variable was collected for residents only and reported as unknown for nonresidents.

^§§^ Reporting form contains only one question for race and ethnicity; therefore, abortions reported for women of Black, White, and other races (Asian and Native American) are not explicitly identified as non-Hispanic.

^¶¶^ Data reported from facilities physically situated in state only.

*** Percentage is based on a total of 361,987 abortions reported among the areas that met reporting standards for race and ethnicity.

^†††^ Number of abortions obtained by women in a specific racial and ethnic group per 1,000 women aged 15–44 years in that same racial and ethnic group. For the total abortion rate only, abortions for women of unknown race and ethnicity were distributed according to the distribution of abortions among women of known race and ethnicity.

^§§§^ Number of abortions obtained by women in a specific racial and ethnic group per 1,000 live births to women in that same racial and ethnic group. For the total abortion ratio only, abortions for women of unknown race and ethnicity were distributed according to the distribution of abortions among women of known race and ethnicity.

For 2022, among the 36 areas that reported by marital status, 12.3% of women who obtained an abortion were married, and 87.7% were unmarried (Table 7). The abortion ratio was 37 abortions per 1,000 live births for married women and 376 abortions per 1,000 live births for unmarried women.

**TABLE 7:** Number and percentage of reported abortions, by known marital status and reporting area of occurrence — selected reporting areas,* United States, 2022

Area	Marital status		Total abortions reported by known marital status
	Married	Unmarried	
	No. (%)^†^	No. (%)	No. (% of all reported abortions)^§^
Alabama	389 (10.3)	3,385 (89.7)	**3,774 (99.9)**
Arizona	1,777 (15.4)	9,753 (84.6)	**11,530 (100.0)**
Arkansas	127 (7.9)	1,484 (92.1)	**1,611 (99.4)**
Colorado	2,237 (17.1)	10,858 (82.9)	**13,095 (92.5)**
Delaware	229 (9.4)	2,216 (90.6)	**2,445 (100.0)**
Florida	9,601 (13.6)	60,888 (86.4)	**70,489 (85.4)**
Georgia	3,839 (10.3)	33,503 (89.7)	**37,342 (96.2)**
Idaho	176 (17.3)	844 (82.7)	**1,020 (99.3)**
Illinois^¶^	4,951 (9.2)	49,018 (90.8)	**53,969 (95.6)**
Indiana	1,268 (13.3)	8,262 (86.7)	**9,530 (100.0)**
Iowa	567 (14.0)	3,490 (86.0)	**4,057 (99.7)**
Kansas	1,746 (14.6)	10,234 (85.4)	**11,980 (97.3)**
Kentucky	310 (12.2)	2,240 (87.8)	**2,550 (100.0)**
Louisiana	397 (8.8)	4,118 (91.2)	**4,515 (98.8)**
Maine	324 (15.3)	1,800 (84.7)	**2,124 (95.5)**
Michigan	3,396 (11.6)	25,792 (88.4)	**29,188 (96.9)**
Minnesota	1,617 (14.3)	9,697 (85.7)	**11,314 (92.9)**
Mississippi	144 (6.3)	2,141 (93.7)	**2,285 (100.0)**
Missouri	33 (37.5)	55 (62.5)	**88 (100.0)**
Montana	313 (18.9)	1,342 (81.1)	**1,655 (97.2)**
Nebraska	331 (13.5)	2,129 (86.5)	**2,460 (96.6)**
Nevada**	1,516 (14.3)	9,082 (85.7)	**10,598 (88.6)**
North Carolina	4,878 (13.2)	32,012 (86.8)	**36,890 (92.1)**
North Dakota	92 (12.3)	659 (87.7)	**751 (99.6)**
Ohio	2,117 (12.3)	15,075 (87.7)	**17,192 (92.9)**
Oklahoma	321 (14.9)	1,835 (85.1)	**2,156 (99.8)**
Rhode Island	180 (13.4)	1,164 (86.6)	**1,344 (97.2)**
South Carolina	936 (12.9)	6,340 (87.1)	**7,276 (100.0)**
South Dakota	24 (17.5)	113 (82.5)	**137 (100.0)**
Tennessee	733 (11.6)	5,601 (88.4)	**6,334 (98.2)**
Texas	2,452 (14.0)	15,061 (86.0)	**17,513 (100.0)**
Utah	831 (21.8)	2,977 (78.2)	**3,808 (99.6)**
Vermont	193 (18.7)	838 (81.3)	**1,031 (93.1)**
Virginia^††,§§^	1,165 (7.0)	15,557 (93.0)	**16,722 (100.0)**
West Virginia	144 (17.3)	687 (82.7)	**831 (100.0)**
Wisconsin	337 (10.2)	2,982 (89.8)	**3,319 (99.6)**
**Total**	**49,691 (12.3)**	**353,232 (87.7)**	**402,923 (94.0)^¶¶^**
Abortion ratio***	**37**	**376**	**NA**

**Abbreviation:** NA = not applicable.

* Data from 36 reporting areas; excludes 16 reporting areas (Alaska, California, Connecticut, District of Columbia, Hawaii, Maryland, Massachusetts, New Hampshire, New Jersey, New Mexico, New York City, New York State, Oregon, Pennsylvania, Washington, and Wyoming) that did not report, did not report by marital status, or did not meet reporting standards.

^†^ Percentages for the individual component categories might not add to 100% because of rounding.

^§^ Percentage is calculated as the number of abortions reported by known marital status divided by the sum of abortions reported by known and unknown marital status. Values ≥99.95% are rounded to 100.0%.

^¶^ Includes residents only.

** For August–December 2022, variable was collected for residents only and reported as unknown for nonresidents.

^††^ Recorded as patient married or not married to father.

^§§^ Data reported from facilities physically situated in state only.

^¶¶^ Percentage is based on a total of 428,577 abortions reported among the areas that met reporting standards for marital status; reporting standards for marital status were applied to abortions for residents of Illinois only.

*** Number of abortions obtained by marital status per 1,000 live births to women of the same marital status. For the total abortion ratio only, abortions for women of unknown marital status were distributed according to the distribution of abortions among women of known marital status.

### Previous Live Births and Previous Induced Abortions

For 2022, among the 41 areas that reported the number of previous live births, 40.6%, 24.1%, 19.5%, 9.4%, and 6.5% of abortions reported were among women who had zero, one, two, three, or four or more previous live births, respectively (Table 8). Among the 41 areas that reported the number of previous induced abortions, 56.1%, 24.7%, 10.9%, and 8.2% of abortions reported were among women who had had zero, one, two, or three or more previous induced abortions, respectively (Table 9).

**TABLE 8:** Number and percentage of reported abortions, by known number of previous live births and reporting area of occurrence — selected reporting areas,* United States, 2022

Area	No. of previous live births					Total abortions reported by known number of previous live births
	0	1	2	3	≥4	
	No. (%)^†^	No. (%)	No. (%)	No. (%)	No. (%)	No. (% of all reported abortions)^§^
Alabama	1,320 (34.9)	1,052 (27.9)	778 (20.6)	401 (10.6)	226 (6.0)	**3,777 (100.0)**
Alaska	587 (47.1)	263 (21.1)	216 (17.3)	100 (8.0)	81 (6.5)	**1,247 (100.0)**
Arizona	5,432 (47.9)	2,359 (20.8)	1,939 (17.1)	917 (8.1)	695 (6.1)	**11,342 (98.4)**
Arkansas	526 (32.4)	440 (27.1)	341 (21.0)	174 (10.7)	140 (8.6)	**1,621 (100.0)**
Colorado	8,302 (59.3)	2,478 (17.7)	1,860 (13.3)	866 (6.2)	488 (3.5)	**13,994 (98.9)**
Delaware	1,025 (41.9)	579 (23.7)	490 (20.0)	207 (8.5)	144 (5.9)	**2,445 (100.0)**
Florida	30,388 (38.1)	20,855 (26.2)	16,485 (20.7)	7,218 (9.1)	4,725 (5.9)	**79,671 (96.5)**
Georgia	15,314 (39.5)	9,444 (24.3)	7,639 (19.7)	3,767 (9.7)	2,648 (6.8)	**38,812 (100.0)**
Hawaii	1,048 (55.0)	314 (16.5)	289 (15.2)	150 (7.9)	103 (5.4)	**1,904 (96.6)**
Idaho	504 (49.4)	222 (21.8)	163 (16.0)	74 (7.3)	57 (5.6)	**1,020 (99.3)**
Indiana	3,642 (38.2)	2,295 (24.1)	1,929 (20.2)	1,007 (10.6)	653 (6.9)	**9,526 (100.0)**
Iowa	1,793 (44.2)	872 (21.5)	735 (18.1)	368 (9.1)	293 (7.2)	**4,061 (99.8)**
Kansas	5,038 (40.9)	2,863 (23.2)	2,397 (19.5)	1,226 (10.0)	793 (6.4)	**12,317 (100.0)**
Kentucky	857 (33.6)	702 (27.5)	559 (21.9)	260 (10.2)	171 (6.7)	**2,549 (100.0)**
Louisiana	1,432 (31.3)	1,203 (26.3)	1,046 (22.9)	524 (11.5)	365 (8.0)	**4,570 (100.0)**
Maine	1,113 (50.4)	465 (21.1)	368 (16.7)	164 (7.4)	97 (4.4)	**2,207 (99.2)**
Massachusetts	7,627 (48.3)	3,648 (23.1)	2,767 (17.5)	1,170 (7.4)	580 (3.7)	**15,792 (88.9)**
Michigan^¶^	10,558 (35.1)	7,627 (25.4)	6,482 (21.5)	3,161 (10.5)	2,256 (7.5)	**30,084 (99.9)**
Minnesota	5,168 (42.5)	2,700 (22.2)	2,293 (18.8)	1,190 (9.8)	821 (6.7)	**12,172 (100.0)**
Mississippi	717 (31.4)	599 (26.2)	518 (22.7)	250 (10.9)	202 (8.8)	**2,286 (100.0)**
Missouri	28 (31.8)	19 (21.6)	17 (19.3)	12 (13.6)	12 (13.6)	**88 (100.0)**
Montana	879 (51.6)	383 (22.5)	256 (15.0)	116 (6.8)	68 (4.0)	**1,702 (100.0)**
Nebraska	1,110 (43.7)	532 (21.0)	467 (18.4)	248 (9.8)	182 (7.2)	**2,539 (99.7)**
Nevada**	5,081 (44.0)	2,517 (21.8)	2,136 (18.5)	1,066 (9.2)	758 (6.6)	**11,558 (96.7)**
New Mexico	4,764 (44.1)	2,357 (21.8)	1,906 (17.7)	1,005 (9.3)	760 (7.0)	**10,792 (92.0)**
North Carolina	13,840 (37.7)	8,790 (23.9)	7,269 (19.8)	3,731 (10.2)	3,113 (8.5)	**36,743 (91.8)**
North Dakota	312 (41.4)	163 (21.6)	144 (19.1)	77 (10.2)	58 (7.7)	**754 (100.0)**
Ohio^††^	6,348 (35.3)	4,683 (26.1)	3,795 (21.1)	1,874 (10.4)	1,269 (7.1)	**17,969 (97.1)**
Oregon	4,602 (53.7)	1,705 (19.9)	1,290 (15.1)	593 (6.9)	375 (4.4)	**8,565 (98.8)**
Pennsylvania	13,143 (37.7)	8,913 (25.6)	7,180 (20.6)	3,425 (9.8)	2,177 (6.2)	**34,838 (100.0)**
Rhode Island	646 (47.1)	334 (24.4)	230 (16.8)	111 (8.1)	50 (3.6)	**1,371 (99.1)**
South Carolina	3,137 (43.1)	1,821 (25.0)	1,320 (18.1)	669 (9.2)	329 (4.5)	**7,276 (100.0)**
South Dakota	58 (42.3)	38 (27.7)	16 (11.7)	17 (12.4)	8 (5.8)	**137 (100.0)**
Tennessee	2,183 (33.8)	1,719 (26.7)	1,386 (21.5)	693 (10.7)	469 (7.3)	**6,450 (100.0)**
Texas	7,083 (40.4)	4,054 (23.1)	3,580 (20.4)	1,778 (10.2)	1,019 (5.8)	**17,514 (100.0)**
Utah	2,012 (52.6)	773 (20.2)	576 (15.1)	266 (7.0)	197 (5.2)	**3,824 (100.0)**
Vermont	645 (58.2)	212 (19.1)	142 (12.8)	68 (6.1)	41 (3.7)	**1,108 (100.0)**
Virginia^§§^	4,823 (28.8)	4,588 (27.4)	3,373 (20.2)	1,954 (11.7)	1,984 (11.9)	**16,722 (100.0)**
Washington	10,184 (50.6)	4,113 (20.4)	3,438 (17.1)	1,526 (7.6)	860 (4.3)	**20,121 (99.6)**
West Virginia	282 (34.0)	229 (27.6)	185 (22.3)	80 (9.6)	54 (6.5)	**830 (99.9)**
Wyoming	264 (48.9)	113 (20.9)	89 (16.5)	45 (8.3)	29 (5.4)	**540 (100.0)**
**Total**	**183,815 (40.6)**	**109,036 (24.1)**	**88,089 (19.5)**	**42,548 (9.4)**	**29,350 (6.5)**	**452,838 (97.7)^¶¶^**

* Data from 41 reporting areas; excludes 11 reporting areas (California, Connecticut, District of Columbia, Illinois, Maryland, New Hampshire, New Jersey, New York City, New York State, Oklahoma, and Wisconsin) that did not report, did not report by number of previous live births, or did not meet reporting standards.

^†^ Percentages for the individual component categories might not add to 100% because of rounding.

^§^ Percentage is calculated as the number of abortions reported by known number of previous live births divided by the sum of abortions reported by known and unknown number of previous live births. Values ≥99.95% are rounded to 100.0%.

^¶^ Recorded as the number of previous pregnancies carried to term.

** For August–December 2022, variable was collected for residents only and reported as unknown for nonresidents.

^††^ Recorded as the number of living children.

^§§^ Data reported from facilities physically situated in state only.

^¶¶^ Percentage is based on a total of 463,579 abortions reported among the areas that met reporting standards for the number of previous live births.

**TABLE 9:** Number and percentage of reported abortions, by known number of previous induced abortions and reporting area of occurrence — selected reporting areas,* United States, 2022

Area	No. of previous induced abortions				Total abortions reported by known number of previous induced abortions
	0	1	2	≥3	
	No. (%)^†^	No. (%)	No. (%)	No. (%)	No. (% of all reported abortions)^§^
Alabama	2,339 (61.9)	909 (24.1)	355 (9.4)	174 (4.6)	**3,777 (100.0)**
Alaska	840 (67.4)	277 (22.2)	91 (7.3)	39 (3.1)	**1,247 (100.0)**
Arizona	7,487 (66.3)	2,649 (23.4)	791 (7.0)	373 (3.3)	**11,300 (98.0)**
Arkansas	1,036 (63.9)	334 (20.6)	132 (8.1)	119 (7.3)	**1,621 (100.0)**
Colorado	10,154 (71.9)	2,789 (19.7)	822 (5.8)	359 (2.5)	**14,124 (99.8)**
Delaware	1,595 (65.3)	547 (22.4)	198 (8.1)	101 (4.1)	**2,441 (99.8)**
Florida	36,777 (46.6)	21,328 (27.0)	11,009 (14.0)	9,776 (12.4)	**78,890 (95.5)**
Georgia	24,020 (61.9)	8,627 (22.2)	3,892 (10.0)	2,269 (5.8)	**38,808 (100.0)**
Hawaii	1,156 (60.1)	462 (24.0)	182 (9.5)	122 (6.3)	**1,922 (97.6)**
Idaho	731 (71.2)	208 (20.3)	67 (6.5)	21 (2.0)	**1,027 (100.0)**
Indiana	6,241 (65.5)	2,130 (22.4)	751 (7.9)	405 (4.3)	**9,527 (100.0)**
Iowa	2,814 (69.3)	847 (20.9)	261 (6.4)	138 (3.4)	**4,060 (99.8)**
Kansas	8,481 (68.9)	2,620 (21.3)	810 (6.6)	405 (3.3)	**12,316 (100.0)**
Kentucky	1,610 (63.2)	583 (22.9)	204 (8.0)	151 (5.9)	**2,548 (99.9)**
Louisiana	2,933 (64.2)	1,119 (24.5)	349 (7.6)	169 (3.7)	**4,570 (100.0)**
Maine	1,435 (65.5)	525 (24.0)	173 (7.9)	57 (2.6)	**2,190 (98.4)**
Massachusetts	8,505 (49.9)	4,835 (28.4)	2,054 (12.1)	1,641 (9.6)	**17,035 (95.9)**
Michigan	15,030 (50.0)	7,390 (24.6)	4,229 (14.1)	3,434 (11.4)	**30,083 (99.9)**
Minnesota	7,500 (61.7)	2,731 (22.5)	1,028 (8.5)	894 (7.4)	**12,153 (99.8)**
Mississippi	1,549 (67.8)	494 (21.6)	175 (7.7)	68 (3.0)	**2,286 (100.0)**
Missouri	73 (83.0)	9 (10.2)	—**	—**	**88 (100.0)**
Montana	860 (50.5)	579 (34.0)	184 (10.8)	79 (4.6)	**1,702 (100.0)**
Nebraska	1,840 (72.5)	481 (19.0)	143 (5.6)	74 (2.9)	**2,538 (99.6)**
Nevada^¶^	6,906 (59.6)	2,661 (23.0)	1,160 (10.0)	853 (7.4)	**11,580 (96.8)**
New Mexico	2,141 (18.8)	6,552 (57.6)	2,044 (18.0)	636 (5.6)	**11,373 (96.9)**
North Carolina	23,053 (63.3)	7,815 (21.5)	3,436 (9.4)	2,101 (5.8)	**36,405 (90.9)**
North Dakota	522 (69.2)	147 (19.5)	47 (6.2)	38 (5.0)	**754 (100.0)**
Ohio	10,888 (60.6)	4,000 (22.3)	1,811 (10.1)	1,260 (7.0)	**17,959 (97.1)**
Oregon	5,638 (66.0)	1,765 (20.7)	687 (8.0)	453 (5.3)	**8,543 (98.5)**
Pennsylvania	18,242 (52.4)	8,480 (24.3)	4,111 (11.8)	4,005 (11.5)	**34,838 (100.0)**
Rhode Island	826 (60.0)	319 (23.2)	128 (9.3)	103 (7.5)	**1,376 (99.5)**
South Carolina	4,792 (65.9)	1,617 (22.2)	586 (8.1)	281 (3.9)	**7,276 (100.0)**
South Dakota	98 (71.5)	26 (19.0)	—**	—**	**137 (100.0)**
Tennessee	4,036 (62.6)	1,600 (24.8)	544 (8.4)	268 (4.2)	**6,448 (99.9)**
Texas	10,692 (61.0)	4,416 (25.2)	1,573 (9.0)	833 (4.8)	**17,514 (100.0)**
Utah	2,978 (77.9)	617 (16.1)	172 (4.5)	57 (1.5)	**3,824 (100.0)**
Vermont	732 (66.1)	243 (21.9)	83 (7.5)	50 (4.5)	**1,108 (100.0)**
Virginia^††^	4,823 (28.8)	4,588 (27.4)	3,373 (20.2)	3,938 (23.5)	**16,722 (100.0)**
Washington	12,308 (61.3)	4,551 (22.7)	1,793 (8.9)	1,426 (7.1)	**20,078 (99.4)**
West Virginia	591 (71.2)	153 (18.4)	55 (6.6)	31 (3.7)	**830 (99.9)**
Wyoming	389 (72.0)	109 (20.2)	31 (5.7)	11 (2.0)	**540 (100.0)**
**Total**	**254,661 (56.1)**	**112,132 (24.7)**	**49,549 (10.9)**	**37,216 (8.2)**	**453,558 (97.8)^§§^**

* Data from 41 reporting areas; excludes 11 reporting areas (California, Connecticut, District of Columbia, Illinois, Maryland, New Hampshire, New Jersey, New York City, New York State, Oklahoma, and Wisconsin) that did not report, did not report by number of previous induced abortions, or did not meet reporting standards.

^†^ Percentages for the individual component categories might not add to 100% because of rounding.

^§^ Percentage is calculated as the number of abortions reported by known number of previous induced abortions divided by the sum of abortions reported by known and unknown number of previous induced abortions. Values ≥99.95% are rounded to 100.0%.

^¶^ For August–December 2022, variable was collected for residents only and reported as unknown for nonresidents.

** Cells with a numerical value in the range of 1–4 and cells that would allow for calculation of these small values have been suppressed.

^††^ Data reported from facilities physically situated in state only.

^§§^ Percentage is based on a total of 463,579 abortions reported among the areas that met reporting standards for the number of previous induced abortions.

### Weeks of Gestation and Method Type

For 2022, among the 41 areas that reported gestational age at the time of abortion, 78.6% of abortions were performed at ≤9 weeks’ gestation, and 92.8% were performed at ≤13 weeks’ gestation (Table 10). Fewer abortions were performed at 14–20 weeks’ gestation (6.1%) or at ≥21 weeks’ gestation (1.1%). Among the 36 reporting areas that provided data every year on gestational age for 2013–2022, the percentage of abortions performed at ≤13 weeks’ gestation changed from 92.0% in 2013 to 92.5% in 2022 (Table 11). However, within this gestational age range, a shift occurred toward earlier gestational ages, with the percentage of abortions performed at ≤6 weeks’ gestation increasing 20% and the percentage of abortions performed at 7–9 weeks’ and 10–13 weeks’ gestation decreasing 7% and 20%, respectively.

**TABLE 10:** Number and percentage of reported abortions, by known weeks of gestation* and reporting area of occurrence — selected reporting areas,^†^ United States, 2022

Area	Weeks of gestation							Total abortions reported by known gestational age
	≤6	7–9	10–13	14–15	16–17	18–20	≥21	
	No. (%)^§^	No. (%)	No. (%)	No. (%)	No. (%)	No. (%)	No. (%)	No. (% of all reported abortions)^¶^
Alabama**	848 (22.5)	1,816 (48.1)	731 (19.4)	171 (4.5)	89 (2.4)	97 (2.6)	23 (0.6)	**3,775 (99.9)**
Alaska	417 (33.4)	518 (41.5)	229 (18.4)	39 (3.1)	43 (3.4)	—^††^	—^††^	**1,247 (100.0)**
Arizona	3,444 (29.9)	5,319 (46.2)	1,885 (16.4)	382 (3.3)	188 (1.6)	172 (1.5)	115 (1.0)	**11,505 (99.8)**
Arkansas**	20 (1.2)	530 (32.7)	702 (43.3)	155 (9.6)	94 (5.8)	106 (6.5)	14 (0.9)	**1,621 (100.0)**
Colorado	4,915 (34.8)	5,829 (41.2)	1,907 (13.5)	341 (2.4)	344 (2.4)	313 (2.2)	488 (3.5)	**14,137 (99.9)**
Delaware	779 (32.3)	1,100 (45.6)	389 (16.1)	92 (3.8)	12 (0.5)	18 (0.7)	24 (1.0)	**2,414 (98.7)**
Florida	28,983 (35.1)	35,418 (42.9)	13,314 (16.1)	2,693 (3.3)	882 (1.1)	742 (0.9)	549 (0.7)	**82,581 (100.0)**
Georgia	23,286 (60.0)	10,582 (27.3)	3,278 (8.4)	630 (1.6)	426 (1.1)	492 (1.3)	125 (0.3)	**38,819 (100.0)**
Hawaii	649 (33.0)	873 (44.4)	273 (13.9)	72 (3.7)	38 (1.9)	28 (1.4)	33 (1.7)	**1,966 (99.8)**
Idaho	399 (39.9)	414 (41.4)	143 (14.3)	37 (3.7)	—^††^	5 (0.5)	—^††^	**1,000 (97.4)**
Indiana	2,332 (24.5)	5,329 (55.9)	1,765 (18.5)	28 (0.3)	19 (0.2)	28 (0.3)	29 (0.3)	**9,530 (100.0)**
Iowa	1,210 (29.8)	1,895 (46.6)	681 (16.8)	113 (2.8)	88 (2.2)	65 (1.6)	12 (0.3)	**4,064 (99.9)**
Kansas	3,165 (25.7)	5,681 (46.1)	2,381 (19.3)	485 (3.9)	282 (2.3)	273 (2.2)	50 (0.4)	**12,317 (100.0)**
Kentucky	746 (29.3)	1,192 (46.7)	399 (15.6)	95 (3.7)	48 (1.9)	59 (2.3)	11 (0.4)	**2,550 (100.0)**
Louisiana	544 (11.9)	1,760 (38.5)	1,647 (36.0)	418 (9.1)	178 (3.9)	23 (0.5)	0 (0.0)	**4,570 (100.0)**
Maine	715 (32.2)	1,075 (48.4)	325 (14.6)	46 (2.1)	36 (1.6)	—^††^	—^††^	**2,221 (99.8)**
Michigan	10,661 (35.5)	12,122 (40.4)	4,369 (14.6)	1,141 (3.8)	669 (2.2)	659 (2.2)	406 (1.4)	**30,027 (99.7)**
Minnesota	4,438 (36.7)	4,839 (40.0)	1,702 (14.1)	413 (3.4)	276 (2.3)	195 (1.6)	227 (1.9)	**12,090 (99.3)**
Mississippi	305 (13.3)	1,327 (58.0)	509 (22.3)	137 (6.0)	7 (0.3)	—^††^	—^††^	**2,286 (100.0)**
Missouri	—^††^	9 (10.2)	—^††^	14 (15.9)	14 (15.9)	24 (27.3)	15 (17.0)	**88 (100.0)**
Montana	877 (52.0)	587 (34.8)	134 (7.9)	52 (3.1)	23 (1.4)	—^††^	—^††^	**1,687 (99.1)**
Nebraska	833 (32.7)	1,031 (40.5)	487 (19.1)	91 (3.6)	61 (2.4)	31 (1.2)	13 (0.5)	**2,547 (100.0)**
Nevada^§§^	4,248 (36.8)	4,755 (41.2)	1,620 (14.1)	343 (3.0)	207 (1.8)	222 (1.9)	133 (1.2)	**11,528 (96.4)**
New Mexico	4,442 (41.9)	3,176 (29.9)	1,526 (14.4)	372 (3.5)	337 (3.2)	322 (3.0)	434 (4.1)	**10,609 (90.4)**
New York City	19,371 (41.8)	17,498 (37.8)	5,383 (11.6)	1,319 (2.8)	815 (1.8)	911 (2.0)	1,054 (2.3)	**46,351 (100.0)**
North Carolina	13,506 (33.8)	17,299 (43.3)	6,246 (15.6)	1,247 (3.1)	906 (2.3)	747 (1.9)	14 (0.0)	**39,965 (99.8)**
North Dakota	274 (36.3)	310 (41.1)	130 (17.2)	32 (4.2)	8 (1.1)	0 (0.0)	0 (0.0)	**754 (100.0)**
Ohio	6,638 (35.9)	7,639 (41.3)	2,653 (14.3)	641 (3.5)	413 (2.2)	408 (2.2)	107 (0.6)	**18,499 (100.0)**
Oklahoma	369 (17.2)	1,011 (47.0)	542 (25.2)	143 (6.7)	58 (2.7)	19 (0.9)	7 (0.3)	**2,149 (99.5)**
Oregon	3,851 (44.5)	3,190 (36.8)	907 (10.5)	199 (2.3)	158 (1.8)	185 (2.1)	171 (2.0)	**8,661 (99.9)**
Rhode Island	559 (40.4)	384 (27.8)	254 (18.4)	82 (5.9)	44 (3.2)	52 (3.8)	8 (0.6)	**1,383 (100.0)**
South Carolina**	1,850 (25.4)	2,732 (37.5)	2,350 (32.3)	305 (4.2)	10 (0.1)	14 (0.2)	15 (0.2)	**7,276 (100.0)**
South Dakota	—^††^	59 (43.1)	—^††^	—^††^	—^††^	0 (0.0)	0 (0.0)	**137 (100.0)**
Tennessee	1,890 (29.3)	2,707 (42.0)	1,422 (22.0)	229 (3.6)	148 (2.3)	47 (0.7)	7 (0.1)	**6,450 (100.0)**
Texas**	17,191 (98.2)	288 (1.6)	7 (0.0)	—^††^	—^††^	12 (0.1)	8 (0.0)	**17,514 (100.0)**
Utah	1,757 (45.9)	1,336 (34.9)	477 (12.5)	100 (2.6)	76 (2.0)	48 (1.3)	30 (0.8)	**3,824 (100.0)**
Vermont	493 (44.7)	399 (36.1)	129 (11.7)	21 (1.9)	23 (2.1)	23 (2.1)	16 (1.4)	**1,104 (99.6)**
Virginia^¶¶^	8,175 (48.9)	5,792 (34.6)	1,856 (11.1)	335 (2.0)	140 (0.8)	198 (1.2)	225 (1.3)	**16,721 (100.0)**
Washington	9,228 (45.8)	7,356 (36.5)	1,992 (9.9)	461 (2.3)	269 (1.3)	319 (1.6)	525 (2.6)	**20,150 (99.8)**
West Virginia	253 (30.5)	374 (45.1)	152 (18.3)	40 (4.8)	9 (1.1)	—^††^	—^††^	**829 (99.8)**
Wyoming	350 (64.9)	181 (33.6)	8 (1.5)	0 (0.0)	0 (0.0)	0 (0.0)	0 (0.0)	**539 (99.8)**
**Total**	**184,042 (40.2)**	**175,732 (38.4)**	**64,961 (14.2)**	**13,518 (3.0)**	**7,444 (1.6)**	**6,892 (1.5)**	**4,896 (1.1)**	457,485 (99.6)***

* Gestational age based on clinician’s estimate (Alaska, Arizona, Colorado, Delaware, Georgia, Hawaii, Idaho, Indiana, Iowa, Kansas, Kentucky, Louisiana, Maine, Michigan, Minnesota, Mississippi, Missouri, Montana, Nebraska, Nevada, New Mexico, New York City, North Carolina, North Dakota, Ohio, Oregon, Rhode Island, South Dakota, Tennessee, Vermont, Washington, West Virginia, and Wyoming); gestational age calculated from the last normal menstrual period (Florida, Oklahoma and Utah); clinician’s estimate of gestation based on estimated date of conception (Virginia); and probable postfertilization age (Alabama, Arkansas, South Carolina, and Texas).

^†^ Data from 41 reporting areas; excludes 11 reporting areas (California, Connecticut, District of Columbia, Illinois, Maryland, Massachusetts, New Hampshire, New Jersey, New York State, Pennsylvania, and Wisconsin) that did not report, did not report by gestational age, or did not meet reporting standards.

^§^ Percentages for the individual component categories might not add to 100% because of rounding.

^¶^ Percentage is calculated as the number of abortions reported by known gestational age divided by the sum of abortions reported by known and unknown gestational age. Values ≥99.95% are rounded to 100.0%.

** Two weeks were added to the probable postfertilization age to provide a corresponding measure to gestational age based on the clinician’s estimate.

^††^ Cells with a numerical value in the range of 1–4 and cells that would allow for calculation of these small values have been suppressed.

^§§^ For August–December 2022, variable was collected for residents only and reported as unknown for nonresidents.

^¶¶^ Data reported from facilities physically situated in state only.

*** Percentage is based on a total of 459,517 abortions reported among the areas that met reporting standards for gestational age.

**TABLE 11:** Percentage of reported abortions, by known weeks of gestation and year — selected reporting areas,* United States, 2013–2022

Weeks of gestation	Year										% Change	
	2013	2014	2015	2016	2017	2018	2019	2020	2021	2022	2021 to 2022	2013 to 2022
**≤13 weeks (%)^†^**	**92.0**	**91.3**	**91.3**	**91.3**	**91.6**	**91.8**	**92.3**	**92.7**	**92.9**	**92.5**	**−0.4**	**0.5**
≤6	34.8	33.6	34.2	34.4	35.3	36.5	37.9	39.6	39.4	41.6	5.6	19.5
7–9	40.2	40.3	40.3	40.7	40.7	40.1	39.9	39.6	39.7	37.3	−6.0	−7.2
10–13	17.0	17.3	16.7	16.3	15.5	15.2	14.4	13.5	13.8	13.6	−1.4	−20.0
**>13 weeks (%)^†^**	**8.0**	**8.7**	**8.7**	**8.7**	**8.4**	**8.2**	**7.7**	**7.3**	**7.1**	**7.5**	**5.6**	**−6.3**
14–15	3.2	3.5	3.4	3.5	3.3	3.3	3.1	3.0	2.9	2.9	0.0	−9.4
16–17	1.8	2.1	2.1	2.1	2.1	2.0	1.8	1.7	1.7	1.7	0.0	−5.6
18–20	1.8	1.9	1.9	1.9	1.9	1.9	1.7	1.6	1.6	1.7	6.2	−5.6
≥21	1.2	1.3	1.3	1.2	1.2	1.1	1.1	1.0	1.0	1.2	20.0	0.0
**Total no.^§^**	**408,958**	**399,156**	**389,411**	**382,097**	**369,992**	**370,647**	**375,636**	**364,626**	**380,753**	**364,311**	**NA**	**NA**

**Abbreviation:** NA = not applicable.

* Data from 36 reporting areas; excludes 16 areas (California, Connecticut, District of Columbia, Florida, Illinois, Maine, Maryland, Massachusetts, New Hampshire, New Jersey, New York State, Pennsylvania, Rhode Island, Tennessee, Wisconsin, and Wyoming) that did not report, did not report by weeks of gestation, or did not meet reporting standards for ≥1 year.

^†^ Percentages for the individual component categories might not add to 100% because of rounding.

^§^ By year, the total number of abortions represents 72.7%–91.4% of all abortions reported to CDC among the areas that met reporting standards for gestational age during 2013–2022.

For 2022, among the 47 areas that reported by method type and included medication abortion on their reporting form, 53.3% of abortions were early medication abortions (a nonsurgical abortion at ≤9 weeks’ gestation), 35.5% were surgical abortions at ≤13 weeks’ gestation, 6.9% were surgical abortions at >13 weeks’ gestation, and 4.3% were medication abortions at >9 weeks’ gestation; other methods, including intrauterine instillation and hysterectomy or hysterotomy, were rare (<0.1%) (Table 12). During 2013−2022, a total of 39 reporting areas (excludes California, the District of Columbia, Florida, Hawaii, Illinois, Louisiana, Maryland, Missouri, New Hampshire, New Jersey, New Mexico, Tennessee, and Wyoming) provided continuous data and included medication abortion on their reporting form. Among these 39 areas, use of early medication abortion increased 4% from 2021 to 2022 (from 52.1% to 54.0% of abortions) and 129% from 2013 to 2022 (from 23.6% to 54.0% of abortions) (data not shown).

**TABLE 12:** Number and percentage of reported abortions, by known method type and reporting area of occurrence — selected reporting areas,* United States, 2022

Area	Surgical^†^			Medication			Intrauterine instillation^§^	Hysterectomy or hysterotomy	Total abortions reported by known method type
	≤13 weeks’ gestation	>13 weeks’ gestation	Unknown gestational age	≤9 weeks’ gestation	>9 weeks’ gestation	Unknown gestational age			
	No. (%)^¶^	No. (%)	No. (%)	No. (%)	No. (%)	No. (%)	No. (%)	No. (%)	No. (% of all reported abortions)**
Alabama^††^	1,210 (32.1)	369 (9.8)	—^§§^	1,967 (52.1)	226 (6.0)	—^§§^	0 (0.0)	0 (0.0)	**3,774 (99.9)**
Alaska	561 (45.0)	81 (6.5)	—^§§^	574 (46.1)	29 (2.3)	0 (0.0)	—^§§^	0 (0.0)	**1,246 (99.9)**
Arizona	4,829 (41.9)	831 (7.2)	8 (0.1)	5,349 (46.4)	494 (4.3)	17 (0.1)	0 (0.0)	0 (0.0)	**11,528 (100.0)**
Arkansas^††^	873 (53.9)	369 (22.8)	0 (0.0)	216 (13.3)	163 (10.1)	0 (0.0)	0 (0.0)	0 (0.0)	**1,621 (100.0)**
Colorado	3,198 (24.9)	790 (6.2)	11 (0.1)	8,118 (63.3)	708 (5.5)	6 (0.0)	—^§§^	—^§§^	**12,833 (90.7)**
Connecticut^¶¶^	NA	NA	3,409 (30.7)	NA	NA	7,701 (69.3)	—^§§^	—^§§^	**11,111 (99.9)**
Delaware	512 (21.2)	125 (5.2)	16 (0.7)	1,563 (64.8)	184 (7.6)	13 (0.5)	0 (0.0)	0 (0.0)	**2,413 (98.7)**
District of Columbia***	1,599 (39.7)	437 (10.9)	0 (0.0)	NA	NA	1,987 (49.4)	0 (0.0)	0 (0.0)	**4,023 (100.0)**
Florida	30,101 (37.6)	4,387 (5.5)	—^§§^	41,469 (51.8)	4,019 (5.0)	0 (0.0)	0 (0.0)	—^§§^	**79,979 (96.8)**
Georgia	9,778 (25.2)	1,664 (4.3)	—^§§^	26,369 (67.9)	1,006 (2.6)	—^§§^	0 (0.0)	0 (0.0)	**38,818 (100.0)**
Hawaii	796 (40.8)	165 (8.5)	—^§§^	934 (47.8)	54 (2.8)	—^§§^	0 (0.0)	0 (0.0)	**1,952 (99.1)**
Idaho	325 (31.6)	41 (4.0)	7 (0.7)	593 (57.7)	41 (4.0)	20 (1.9)	0 (0.0)	0 (0.0)	**1,027 (100.0)**
Indiana	4,419 (46.4)	64 (0.7)	0 (0.0)	4,895 (51.4)	152 (1.6)	0 (0.0)	0 (0.0)	0 (0.0)	**9,530 (100.0)**
Iowa	808 (19.9)	269 (6.6)	—^§§^	2,708 (66.6)	278 (6.8)	—^§§^	0 (0.0)	0 (0.0)	**4,066 (100.0)**
Kansas	3,888 (31.6)	942 (7.6)	0 (0.0)	6,599 (53.6)	888 (7.2)	0 (0.0)	0 (0.0)	0 (0.0)	**12,317 (100.0)**
Kentucky	1,081 (42.4)	212 (8.3)	0 (0.0)	1,253 (49.1)	—^§§^	0 (0.0)	0 (0.0)	—^§§^	**2,550 (100.0)**
Louisiana	2,135 (47.0)	617 (13.6)	0 (0.0)	1,635 (36.0)	160 (3.5)	0 (0.0)	0 (0.0)	0 (0.0)	**4,547 (99.5)**
Maine	709 (31.9)	102 (4.6)	—^§§^	1,275 (57.4)	130 (5.9)	—^§§^	0 (0.0)	0 (0.0)	**2,220 (99.8)**
Massachusetts^†††^	NA	NA	8,462 (47.7)	NA	NA	9,276 (52.3)	—^§§^	—^§§^	**17,742 (99.9)**
Michigan	11,590 (38.6)	2,783 (9.3)	46 (0.2)	14,489 (48.2)	1,083 (3.6)	34 (0.1)	—^§§^	—^§§^	**30,031 (99.7)**
Minnesota	3,652 (30.0)	1,100 (9.0)	29 (0.2)	6,802 (55.9)	534 (4.4)	56 (0.5)	—^§§^	—^§§^	**12,174 (100.0)**
Mississippi	238 (10.4)	144 (6.3)	0 (0.0)	1,622 (71.0)	280 (12.3)	0 (0.0)	0 (0.0)	0 (0.0)	**2,284 (99.9)**
Missouri	—^§§^	46 (54.8)	—^§§^	0 (0.0)	16 (19.0)	0 (0.0)	0 (0.0)	—^§§^	**84 (95.5)**
Montana	311 (18.3)	88 (5.2)	—^§§^	1,219 (71.6)	69 (4.1)	11 (0.6)	—^§§^	0 (0.0)	**1,702 (100.0)**
Nebraska	449 (17.6)	193 (7.6)	0 (0.0)	1,725 (67.8)	178 (7.0)	0 (0.0)	0 (0.0)	0 (0.0)	**2,545 (99.9)**
Nevada^§§§^	4,596 (41.7)	436 (4.0)	14 (0.1)	5,503 (49.9)	328 (3.0)	143 (1.3)	0 (0.0)	0 (0.0)	**11,020 (92.2)**
New Mexico	3,848 (36.7)	1,071 (10.2)	218 (2.1)	4,522 (43.1)	530 (5.0)	296 (2.8)	—^§§^	—^§§^	**10,497 (89.5)**
New York	8,231 (11.5)	1,922 (2.7)	28,821 (40.3)	7,799 (10.9)	1,807 (2.5)	22,856 (31.9)	110 (0.2)	16 (0.0)	**71,562 (98.4)**
New York City	NA	NA	26,988 (58.2)	NA	NA	19,271 (41.6)	98 (0.2)	16 (0.0)	**46,373 (100.0)**
New York State	8,231 (32.7)	1,922 (7.6)	1,833 (7.3)	7,799 (31.0)	1,807 (7.2)	3,585 (14.2)	12 (0.0)	0 (0.0)	**25,189 (95.6)**
North Carolina	10,040 (25.4)	2,817 (7.1)	39 (0.1)	24,598 (62.3)	1,967 (5.0)	17 (0.0)	—^§§^	—^§§^	**39,481 (98.6)**
North Dakota	328 (43.5)	31 (4.1)	0 (0.0)	361 (47.9)	34 (4.5)	0 (0.0)	0 (0.0)	0 (0.0)	**754 (100.0)**
Ohio	7,801 (42.2)	1,546 (8.4)	—^§§^	9,069 (49.0)	78 (0.4)	0 (0.0)	0 (0.0)	—^§§^	**18,497 (100.0)**
Oklahoma	515 (24.5)	222 (10.6)	—^§§^	1,171 (55.8)	187 (8.9)	—^§§^	—^§§^	0 (0.0)	**2,100 (97.2)**
Oregon	2,420 (28.2)	657 (7.7)	—^§§^	5,159 (60.2)	305 (3.6)	10 (0.1)	—^§§^	15 (0.2)	**8,567 (98.8)**
Pennsylvania^¶¶^	NA	NA	15,715 (45.3)	NA	NA	19,011 (54.7)	0 (0.0)	0 (0.0)	**34,726 (99.7)**
Rhode Island	934 (67.6)	181 (13.1)	—^§§^	257 (18.6)	8 (0.6)	0 (0.0)	0 (0.0)	—^§§^	**1,381 (99.9)**
South Carolina^††^	1,718 (23.6)	334 (4.6)	0 (0.0)	3,835 (52.7)	1,382 (19.0)	0 (0.0)	7 (0.1)	0 (0.0)	**7,276 (100.0)**
South Dakota	85 (62.0)	—^§§^	—^§§^	43 (31.4)	—^§§^	0 (0.0)	0 (0.0)	0 (0.0)	**137 (100.0)**
Tennessee	2,352 (36.5)	423 (6.6)	—^§§^	3,267 (50.7)	401 (6.2)	0 (0.0)	0 (0.0)	—^§§^	**6,445 (99.9)**
Texas^††^	5,598 (32.0)	—^§§^	0 (0.0)	11,887 (67.9)	25 (0.1)	0 (0.0)	0 (0.0)	—^§§^	**17,513 (100.0)**
Utah	1,282 (33.6)	220 (5.8)	—^§§^	2,216 (58.0)	92 (2.4)	0 (0.0)	7 (0.2)	—^§§^	**3,820 (99.9)**
Vermont	266 (24.1)	78 (7.1)	—^§§^	705 (63.9)	51 (4.6)	—^§§^	0 (0.0)	0 (0.0)	**1,104 (99.6)**
Virginia^¶¶¶^	6,217 (37.3)	875 (5.3)	0 (0.0)	9,205 (55.2)	361 (2.2)	—^§§^	0 (0.0)	—^§§^	**16,661 (99.6)**
Washington	5,792 (29.1)	1,515 (7.6)	—^§§^	12,082 (60.7)	458 (2.3)	37 (0.2)	—^§§^	0 (0.0)	**19,889 (98.5)**
West Virginia	378 (45.5)	49 (5.9)	—^§§^	366 (44.1)	35 (4.2)	—^§§^	0 (0.0)	0 (0.0)	**830 (99.9)**
Wisconsin^¶¶,^****	NA	NA	2,100 (64.0)	NA	NA	1,179 (36.0)	0 (0.0)	0 (0.0)	**3,279 (100.0)**
Wyoming	—^§§^	0 (0.0)	0 (0.0)	531 (98.3)	8 (1.5)	—^§§^	0 (0.0)	0 (0.0)	**540 (100.0)**
**Total**	**194,835 (35.5)**	**37,766 (6.9)**	**—^††††^**	**291,980 (53.3)**	**23,412 (4.3)**	**—^§§§§^**	**138 (0.0)**	**65 (0.0)**	**548,196 (98.4)^¶¶¶¶^**

**Abbreviation:** NA = not applicable.

* Data from 47 reporting areas; excludes five reporting areas (California, Illinois, Maryland, New Hampshire, and New Jersey) that did not report, did not report by method type, or did not meet reporting standards. Areas reporting by method type with unknown gestational age or gestational age reported was not compatible with categorizations presented in this table are included.

^†^ Includes uterine aspiration (might also be called dilation and curettage, aspiration curettage, suction curettage, manual vacuum aspiration, menstrual extraction, or sharp curettage) and dilation and evacuation procedures.

^§^ Intrauterine instillation reported at ≤12 weeks’ gestation is considered as unknown for method type.

^¶^ Percentages for the individual component categories might not add to 100% because of rounding.

** Percentage is calculated as the number of abortions reported by known method type divided by the sum of abortions reported by known and unknown method type. Values ≥99.95% are rounded to 100.0%.

^††^ Two weeks were added to the probable postfertilization age to provide a corresponding measure to gestational age based on the clinician’s estimate.

^§§^ Cells with a numerical value in the range of 1–4 and cells that would allow for calculation of these small values have been suppressed.

^¶¶^ Numbers for surgical abortions ≤13 weeks’ and >13 weeks’ gestation and medication abortions ≤9 weeks’ versus >9 weeks’ gestation are not presented because gestational age reported was not compatible with these categorizations.

*** Gestational age is based on clinician’s estimate. Numbers for medication abortions at ≤9 weeks versus >9 weeks are not presented because gestational age reported was not compatible with these categorizations.

^†††^ Numbers for surgical abortions at ≤13 weeks’ versus >13 weeks’ gestation and for medication abortions at ≤9 weeks’ versus >9 weeks’ gestation are not presented because gestational age data were not provided by method type.

^§§§^ For August–December 2022, variable was collected for residents only and reported as unknown for nonresidents.

^¶¶¶^ Data reported from facilities physically situated in state only.

**** Includes residents only. Wisconsin reports as surgical, unspecified and does not differentiate surgical abortions from hysterectomy or hysterotomy. All abortions were reported as surgical or chemically induced. For this report, all surgical abortions were classified as surgical and all chemical abortions as medication.

^††††^ For the total only, surgical abortions reported without a gestational age were distributed among the surgical abortion categories according to the distribution of surgical abortions at known gestational age.

^§§§§^ For the total only, medication abortions reported without a gestational age were distributed among the medication abortion categories according to the distribution of medication abortions at known gestational age.

^¶¶¶¶^ Percentage is based on a total of 556,872 abortions reported among the areas that met reporting standards for method type.

Among the 40 areas that reported abortions categorized by weeks of gestation and method type for 2022, surgical abortion accounted for the highest percentage of abortions at >10 weeks’ gestation (Table 13). Surgical abortion accounted for 25.2% of abortions at ≤6 weeks’ gestation, 34.4% of abortions at 7–9 weeks’ gestation, 72.4% of abortions at 10–13 weeks’ gestation, 94.9%–98.1% of abortions at 14–20 weeks’ gestation, and 80.0% of abortions at ≥21 weeks’ gestation. In contrast, medication abortion accounted for 74.7% of abortions at ≤6 weeks’ gestation, 65.6% of abortions at 7–9 weeks’ gestation, 27.6% of abortions at 10–13 weeks’ gestation, 1.9%–4.8% of abortions at 14–20 weeks’ gestation, and 19.4% of abortions at ≥21 weeks’ gestation. For each gestational age category as applicable, abortions performed by intrauterine instillation or hysterectomy or hysterotomy were rare (<0.1%–0.4% of abortions).

**TABLE 13:** Number and percentage of reported abortions, by known weeks of gestation and method type — selected reporting areas,* United States, 2022

Method type	Weeks of gestation							Total
	≤6	7–9	10–13	14–15	16–17	18–20	≥21	
	No. (%)^†^	No. (%)	No. (%)	No. (%)	No. (%)	No. (%)	No. (%)	No. (%)
**Surgical^§^**								
≤13 weeks’ gestation	41,971 (25.2)	57,516 (34.4)	45,997 (72.4)	NA	NA	NA	NA	**145,484 (34.1)**
>13 weeks’ gestation	NA	NA	NA	12,446 (98.1)	6,900 (97.7)	5,902 (94.9)	2,952 (80.0)	**28,200 (6.6)**
**Medication^¶^**								
≤9 weeks’ gestation	124,263 (74.7)	109,687 (65.6)	NA	NA	NA	NA	NA	**233,950 (54.8)**
>9 weeks’ gestation	NA	NA	17,548 (27.6)	237 (1.9)	159 (2.3)	299 (4.8)	717 (19.4)	**18,960 (4.4)**
**Intrauterine instillation**	—**	—**	0 (0.0)	1 (0.0)	3 (0.0)	9 (0.1)	16 (0.4)	**29 (0.0)**
**Hysterectomy or hysterotomy**	16 (0.0)	15 (0.0)	4 (0.0)	1 (0.0)	1 (0.0)	8 (0.1)	3 (0.1)	**48 (0.0)**
**Total**	**166,250 (100.0)**	**167,218 (100.0)**	**63,549 (100.0)**	**12,685 (100.0)**	**7,063 (100.0)**	**6,218 (100.0)**	**3,688 (100.0)**	**426,671 (100.0)**

**Abbreviation:** NA = not applicable.

* Data from 40 reporting areas; excludes 12 reporting areas (California, Connecticut, District of Columbia, Illinois, Maryland, Massachusetts, New Hampshire, New Jersey, New York City, New York State, Pennsylvania, and Wisconsin) that did not report, did not report by weeks of gestation, did not meet reporting standards, or did not have medication abortion as a specific category on their reporting form.

^†^ For each gestational age category, percentages of all method types might not add to 100% because of rounding.

^§^ Includes uterine aspiration (might also be called dilation and curettage, aspiration curettage, suction curettage, manual vacuum aspiration, menstrual extraction, or sharp curettage) and dilation and evacuation procedures.

^¶^ The administration of medication or medications to induce an abortion; at ≤9 weeks’ gestation, typically involves the use of mifepristone and misoprostol and at >9 weeks’ gestation, typically involves the use of vaginal prostaglandins.

** Intrauterine instillations reported at ≤12 weeks’ gestation have not been included with known values.

### Weeks of Gestation by Age Group and Race and Ethnicity

In reporting areas that provided data that met CDC reporting standards, abortions that were categorized by weeks of gestation were further categorized by age and by race and ethnicity (Table 14). In every subgroup for these characteristics, the highest percentage of abortions occurred at ≤9 weeks’ gestation. In 40 reporting areas, by age, 56.3% of adolescents aged <15 years and 72.5% of adolescents aged 15–19 years obtained an abortion at ≤9 weeks’ gestation, compared with ≥77.7% for women aged ≥20 years. Conversely, 18.3% of adolescents aged <15 years and 9.6% of adolescents aged 15–19 years obtained an abortion after 13 weeks’ gestation, compared with 6.2%–7.6% for women aged ≥20 years. In 30 reporting areas, by race and ethnicity, 77.1% of abortions obtained by Black women occurred at ≤9 weeks’ gestation, compared with 79.4% by non-Hispanic women in the other race category, 80.5% by White women, and 81.8% by Hispanic women. Conversely, 5.3% of abortions obtained by Hispanic women occurred after 13 weeks’ gestation, compared with 6.0% by White women, 6.8% by Black women, and 7.2% by non-Hispanic women in the other race category.

**TABLE 14:** Number and percentage of reported abortions, by known weeks of gestation, age group, and race and ethnicity — selected reporting areas, United States, 2022

Characteristic	Weeks of gestation							Total
	≤6	7–9	10–13	14–15	16–17	18–20	≥21	
	No. (%)	No. (%)	No. (%)	No. (%)	No. (%)	No. (%)	No. (%)	No. (%)
Age group, yrs*^,†^								
<15	191 (21.5)	309 (34.8)	226 (25.5)	60 (6.8)	34 (3.8)	39 (4.4)	29 (3.3)	**888 (100.0)**
15–19	11,452 (33.7)	13,174 (38.8)	6,019 (17.7)	1,324 (3.9)	784 (2.3)	694 (2.0)	492 (1.4)	**33,939 (100.0)**
20–24	45,505 (38.6)	46,113 (39.1)	18,079 (15.3)	3,619 (3.1)	1,945 (1.6)	1,668 (1.4)	979 (0.8)	**117,908 (100.0)**
25–29	47,138 (40.8)	45,201 (39.1)	16,039 (13.9)	3,150 (2.7)	1,644 (1.4)	1,497 (1.3)	937 (0.8)	**115,606 (100.0)**
30–34	34,775 (41.7)	31,674 (38.0)	11,474 (13.8)	2,321 (2.8)	1,210 (1.5)	1,122 (1.3)	786 (0.9)	**83,362 (100.0)**
35–39	18,385 (42.4)	16,110 (37.2)	5,784 (13.3)	1,250 (2.9)	707 (1.6)	676 (1.6)	447 (1.0)	**43,359 (100.0)**
≥40	6,695 (46.0)	5,043 (34.7)	1,711 (11.8)	422 (2.9)	270 (1.9)	252 (1.7)	154 (1.1)	**14,547 (100.0)**
**Total**	**164,141 (40.1)**	**157,624 (38.5)**	**59,332 (14.5)**	**12,146 (3.0)**	**6,594 (1.6)**	**5,948 (1.5)**	**3,824 (0.9)**	**409,609 (100.0)**
Race and ethnicity*^,§^								
Non-Hispanic								
Black	47,820 (38.2)	48,681 (38.9)	20,098 (16.1)	4,060 (3.2)	1,942 (1.6)	1,718 (1.4)	730 (0.6)	**125,049 (100.0)**
White	41,789 (42.1)	38,105 (38.4)	13,338 (13.4)	2,615 (2.6)	1,419 (1.4)	1,314 (1.3)	723 (0.7)	**99,303 (100.0)**
Other^¶^	9,668 (42.5)	8,394 (36.9)	3,063 (13.5)	651 (2.9)	353 (1.6)	370 (1.6)	248 (1.1)	**22,747 (100.0)**
Hispanic	28,837 (43.4)	25,529 (38.4)	8,592 (12.9)	1,603 (2.4)	789 (1.2)	764 (1.1)	379 (0.6)	**66,493 (100.0)**
**Total**	**128,114 (40.9)**	**120,709 (38.5)**	**45,091 (14.4)**	**8,929 (2.8)**	**4,503 (1.4)**	**4,166 (1.3)**	**2,080 (0.7)**	**313,592 (100.0)**

* Percentages for the individual component categories might not add to 100% because of rounding.

^†^ Data from 40 reporting areas; excludes 12 reporting areas (California, Connecticut, District of Columbia, Illinois, Maryland, Massachusetts, New Hampshire, New Jersey, New York City, New York State, Pennsylvania, and Wisconsin) that did not report, did not report weeks of gestation by age, or did not meet reporting standards.

^§^ Data from 30 reporting areas; excludes 22 reporting areas (Arkansas, California, Colorado, Connecticut, District of Columbia, Hawaii, Illinois, Iowa, Louisiana, Maryland, Massachusetts, Nebraska, New Hampshire, New Jersey, New Mexico, New York City, New York State, Ohio, Oklahoma, Pennsylvania, Washington, and Wisconsin) that did not report, did not report weeks of gestation by race and ethnicity, or did not meet reporting standards.

¶ Including Alaska Native, American Indian, Asian (Indian, Chinese, Filipino, Japanese, Korean, Vietnamese, or other Asian), Native Hawaiian or other Pacific Islander (Native Hawaiian, Guamanian or Chamorro, Samoan, or other Pacific Islander), other races, and multiple races.

### Abortion Mortality

Using national PMSS data (*37*), CDC identified five abortion-related deaths for 2021, the most recent year for which data were reviewed for abortion-related deaths (Table 15). Investigation of these cases indicated all deaths were related to legal abortion.

**TABLE 15:** Number of deaths and case-fatality rates* for abortion-related deaths reported to CDC, by type of abortion — United States, 1973–2021^†^

Year	Type of abortion			Total deaths	CFR per 100,000 legal abortions
	Induced		Unknown^¶^		
	Legal^§^	Illegal			
**1973–1977**					**2.09**
1973	25	19	3	**47**	
1974	26	6	1	**33**	
1975	29	4	1	**34**	
1976	11	2	1	**14**	
1977	17	4	0	**21**	
**1978–1982**					**0.78**
1978	9	7	0	**16**	
1979	22	0	0	**22**	
1980	9	1	2	**12**	
1981	8	1	0	**9**	
1982	11	1	0	**12**	
**1983–1987**					**0.66**
1983	11	1	0	**12**	
1984	12	0	0	**12**	
1985	11	1	1	**13**	
1986	11	0	2	**13**	
1987	7	2	0	**9**	
**1988–1992**					**0.74**
1988	16	0	0	**16**	
1989	12	1	0	**13**	
1990	9	0	0	**9**	
1991	11	1	0	**12**	
1992	10	0	0	**10**	
**1993–1997**					**0.52**
1993	6	1	2	**9**	
1994	10	2	0	**12**	
1995	4	0	0	**4**	
1996	9	0	0	**9**	
1997	7	0	0	**7**	
**1998–2002**					**0.63**
1998	9	0	0	**9**	
1999	4	0	0	**4**	
2000	11	0	0	**11**	
2001	7	1	0	**8**	
2002	10	0	0	**10**	
**2003–2007**					**0.60**
2003	10	0	0	**10**	
2004	7	1	0	**8**	
2005	7	0	0	**7**	
2006	7	0	0	**7**	
2007	6	0	0	**6**	
**2008–2012**					**0.65**
2008	12	0	0	**12**	
2009	8	0	0	**8**	
2010	10	0	0	**10**	
2011	2	0	0	**2**	
2012	4	0	0	**4**	
**2013–2021**					**0.46**
2013	4	0	0	**4**	
2014	6	0	0	**6**	
2015	2	0	1	**3**	
2016	6	1	1	**8**	
2017	3	0	0	**3**	
2018	2	0	0	**2**	
2019	4	0	0	**4**	
2020	6	0	0	**6**	
2021	5	0	0	**5**	

**Abbreviation:** CFR = case-fatality rate.

* Number of legal induced abortion–related deaths per 100,000 reported legal induced abortions. Because a substantial number of legal induced abortions occurred outside reporting areas that provided data to CDC, national CFRs (i.e., number of legal induced abortion–related deaths per 100,000 reported legal induced abortions in the United States) were calculated with denominator data from the Guttmacher Institute’s national survey of abortion-providing facilities; for 2021, denominator data for 2020 were used, the most recent year for which data are available. CFRs were computed for consecutive 5-year periods during 1973–2012 and then for a consecutive 9-year period during 2013–2021 because rates based on <20 cases might be unstable.

^†^ Certain numbers might differ from those in reports published previously because additional information has been supplied to CDC after publication.

^§^ An abortion is defined as legal if it was performed by a licensed clinician within the limits of state or jurisdiction law.

^¶^ Unknown whether abortion was induced or spontaneous.

The annual number of deaths related to legal induced abortion has fluctuated from year to year since 1973 (Table 15). During 2013–2021, the national case-fatality rate for legal induced abortion was 0.46 deaths related to legal induced abortions per 100,000 reported legal abortions. This case-fatality rate was lower than the rates for the previous 5-year periods.

## Discussion

For 2022, a total of 613,383 abortions were reported to CDC by 48 areas. Among the 47 continuously reporting areas, the abortion rate was 11.2 abortions per 1,000 women aged 15–44 years, and the abortion ratio was 199 abortions per 1,000 live births. From 2021 to 2022, the number of abortions decreased 2%, the abortion rate decreased 3%, and the abortion ratio decreased 2%. Multiple factors influence measures of abortion, including access to health care services and contraception (*40**–**42*), the availability of abortion providers and clinics (*38**,**43**–**47*), changes in legal restrictions and regulations (*48**–**56*), parental involvement laws (*57**,**58*), and economic considerations that influence family planning decisions and contraceptive use (*59**,**60*).

Abortion measures differ by age group. Among areas that reported data continuously by age during 2013–2022, women aged 20–29 years accounted for the highest percentages of abortions and had the highest abortion rates, whereas adolescents aged <15 years accounted for the lowest percentage of abortions and had the lowest abortion rate and adolescents aged <15 years and 15–19 years had the highest abortion ratios. During 2013–2022, women aged ≥40 years accounted for a small percentage of reported abortions (≤3.7%). However, the abortion ratio among women aged ≥40 years continues to be higher than among women aged 25–39 years.

The percentage changes in adolescent abortions described in this report are important for monitoring changes in adolescent pregnancies in the United States. From 2013 to 2022, national birth data indicate that the birth rate for adolescents aged 15–19 years decreased 49% (*35*), and the findings in this report indicate that the abortion rate for the same age group decreased 34%, suggesting a decrease in adolescent pregnancies.

The factors leading to higher abortion rates among certain racial and ethnic minority groups are complex. Race and ethnicity as described in this report are markers not drivers of differences in abortion rates. In addition to disparities in rates of unintended pregnancies (*61*), structural factors, including unequal access to quality family planning services (*62*), economic inequities, and mistrust of the medical system (*63*), can contribute to observed differences. As in previous years, abortion rates and ratios differed across racial and ethnic groups. In 2022, compared with White women, abortion rates and ratios were 4.3 and 4.0 times higher among Black women and 2.0 and 1.6 times higher among Hispanic women. Similar differences by race and ethnicity have been demonstrated in other U.S.-based studies (*2**,**10**–**13*).

During 2022, approximately four of five abortions occurred early in gestation (≤9 weeks), when the risks for complications are lowest (*64**–**67*). During the past 10 years, this percentage increased from 75.0% in 2013 to 78.9% in 2022. Moreover, among areas that reported abortions at ≤13 weeks’ gestation by individual week, the distribution of abortions by gestational age continued to shift toward earlier weeks of gestation, with the percentage of early abortions performed at ≤6 weeks’ gestation increasing from 34.8% in 2013 to 41.6% in 2022. Previous research indicates that the distribution of abortions by gestational age differs by various sociodemographic characteristics (*68**–**70*). In this report, the percentage of adolescents aged ≤19 years who obtained abortions at >13 weeks’ gestation was higher than the percentage of women aged ≥20 years. The gestational age when abortions are performed can be influenced by multiple factors, including state or jurisdiction abortion restrictions, accurate estimation of gestational age, income level, age, and presence of pregnancy-related health conditions (*48**,**67**,**69**–**74*).

Changes in clinical practices have facilitated the trend of obtaining abortions earlier in pregnancy. Research conducted in the United States during the 1970s indicated that surgical abortion procedures performed at ≤6 weeks’ gestation, compared with 7–12 weeks’ gestation, were less likely to result in successful termination of the pregnancy (*75*). However, subsequent advances in technology (e.g., improved transvaginal ultrasonography and sensitivity of pregnancy tests) have allowed early surgical abortions to be performed with completion rates exceeding 97% (*76**–**79*). Likewise, the development of early medication abortion regimens has allowed for abortions to be performed early in gestation, with completion rates for regimens that combine mifepristone and misoprostol reaching 96%–98% (*79**–**83*).

Trends for early medication abortions are reported to monitor any changes in clinical practice that might have occurred with the accumulation of evidence on the safety and effectiveness of medication abortion beyond 63 days of gestation (8 completed weeks’ gestation) (*84*), changes in professional practice guidelines (*85**,**86*), and the 2016 FDA extension of the gestational age limit for the use of mifepristone for early medication abortion from 63 days to 70 days (9 completed weeks’ gestation) (*87*). Among abortions occurring at ≤9 weeks’ gestation in 2022, approximately two thirds (70.2%) were reported as early medication abortions. In 2022, the most common method among abortions reported overall was early medication abortion at ≤9 weeks’ gestation (53.3%). Among continuously reporting areas that reported by method type and included medication abortion on their reporting form, the percentage of all abortions performed by early medication abortion increased 129% from 2013 to 2022 and increased 4% from 2021 to 2022.

Because the annual number of deaths related to legal induced abortion is small and statistically unstable, case-fatality rates were calculated for consecutive 5-year periods during 1973–2012 and then for a consecutive 9-year period during 2013–2021. The national case-fatality rate for legal induced abortion was 0.46 per 100,000 abortions during 2013–2021. Since 1978, all rates for the preceding 5-year periods have been <1 death per 100,000 abortions, demonstrating the low risk for death associated with legal induced abortion.

## Limitations

The findings in this report are subject to at least five limitations. First, because reporting to CDC is voluntary and reporting requirements vary by the individual reporting areas (*16**,**17*), CDC is unable to report the total number of abortions performed in the United States. Of the 52 areas from which CDC requested data for 2022, California, Maryland, New Hampshire, and New Jersey did not submit abortion data. In 2020, the most recent year for which data are available through the Guttmacher Institute’s national survey of abortion-providing facilities, abortions performed in these states accounted for approximately 20% of all abortions in the United States (*38*). CDC receives aggregated data from the central health agencies of reporting areas, which might result in different estimates than reported by the Guttmacher Institute. Completeness of reporting might vary by reporting area. Whereas most reporting areas have a legal requirement to submit a report for every abortion performed (*16*), adherence to this requirement might vary (*17*). Access to telehealth services for abortion varies by state or jurisdiction (*47*) and reporting of abortions that occur using telehealth services also might vary by reporting area.

Second, many states and jurisdictions use abortion reporting forms that differ from the technical guidance that NAPHSIS developed with technical assistance from CDC. Consequently, certain reporting areas do not collect all variables requested by CDC (e.g., race and ethnicity), do not collect or report all variables for out-of-area residents (e.g., Nevada), or do not report the data in a manner consistent with this guidance (e.g., gestational age). Missing demographic information can reduce the extent to which the statistics in this report represent women who have had abortions. Only 32 reporting areas reported race and ethnicity data to CDC that met CDC’s reporting standards. Certain areas that either do not report to CDC (e.g., California) or do not report race and ethnicity data (e.g., Illinois) have sufficiently large populations of racial and ethnic minority groups that the absence of data from these areas reduces the representativeness of CDC data for these variables. In addition, because of the variability in data collection for race and ethnicity among reporting areas, data for specific racial and ethnic groups beyond Black, White, and Hispanic are not requested or reported. In addition, certain areas collect gestational age data that are based on estimated date of conception or probable postfertilization age, which are not consistent with medical conventions for gestational age reporting. Without medical guidance on how to report these data, the validity and reliability of gestational age for these reporting areas is uncertain.

Third, abortion data are compiled and reported to CDC by the central health agency of the reporting area in which the abortion was performed rather than the reporting area in which the person lived. Thus, the available population (*24**–**33*) and birth data (*34**,**35*), which are organized by the states or jurisdictions in which women live, might differ from the population of women who undergo abortions in a specific reporting area. This likely results in an overestimation of abortion measures for reporting areas in which a higher percentage of abortions are obtained by out-of-area residents and an underestimation of abortions for reporting areas where residents more frequently obtain abortions out of area. Limited abortion services, stringent regulatory requirements for obtaining an abortion, or geographic proximity to services in another state or jurisdiction might influence where women obtain abortion services (*88**,**89*). Studies have demonstrated an increase in out-of-area abortions for residents of states with legal restrictions for abortion (*53**,**90*).

Fourth, in this report, the presentations of percentage change for abortions by variables (e.g., weeks of gestation) are limited to the aggregate of the 47 jurisdictions that met reporting standards for every year during 2013–2022. This approach allows for assessment of changes in continuously reporting areas; however, specific jurisdictions’ patterns might differ from aggregate 10-year or year-to-year comparisons.

Finally, CDC reporting of sociodemographic characteristics of women obtaining abortions is limited to data collected on state or jurisdiction reporting forms. Therefore, the examination of additional demographic variables (e.g., income and education) is not possible.

## Public Health Implications

Ongoing surveillance of legal induced abortion is important for three reasons. First, abortion surveillance can be used to help evaluate programs aimed at promoting equitable access to patient-centered contraceptive care in the United States to reduce unintended pregnancies. Up to 42% of pregnancies in the United States are unintended (*61*), and use of effective contraception is a strategy to reduce unintended pregnancy (*91*). Efforts to improve contraceptive access that consider patient perspectives and remove barriers (e.g., cost) have been associated with declines in the rate of abortion (*40**–**42*). Providing contraception at low or no cost can increase contraceptive access and use (*92**–**94*). Improvements in provider reimbursement and training, patient-centered counseling, youth-friendly services, and client awareness of available contraceptive methods also might help to improve contraceptive access (*95**–**97*), ensure equitable access to patient-centered contraceptive care, and promote equitable reproductive health in the United States (*98*). Second, routine abortion surveillance can be used to assess changes in clinical practice patterns over time. Information in this report on the number of abortions performed through different methods (e.g., medication or surgical) and at different gestational ages provides the denominator data that are necessary for analyses of the relative safety of abortion practices (*99*). Finally, information on the number of pregnancies ending in abortion is used in conjunction with data on births and fetal losses to estimate the number of pregnancies in the United States and determine rates for various outcomes of public health importance (*61*).
